# Understanding the Beneficial Role of Transition-Metal Layer Na^+^ Substitution on the Structure and Electrochemical Properties of the P2-Layered Cathode Na_2+*x*_Ni_2–*x*/2_TeO_6_

**DOI:** 10.1021/acs.chemmater.4c02798

**Published:** 2025-03-13

**Authors:** Nicholas S. Grundish, Graeme Henkelman, John B. Goodenough, Claude Delmas, Dany Carlier, Ieuan D. Seymour

**Affiliations:** 1 Materials Science and Engineering Program and Texas Materials Institute, University of Texas, Austin, Texas 78712, United States; 2 Department of Chemistry and Oden Institute for Computational Engineering and Sciences, The University of Texas at Austin, Austin, Texas 78712, United States; 3 Univ. Bordeaux, CNRS, Bordeaux INP, ICMCB, UMR 5026, Pessac F-33600, France; 4 RS2E, Réseau Français sur le Stockage Electrochimique de l’Energie, FR CNRS 3459, Cedex 1, Amiens F-80039, France; 5 Advanced Centre for Energy and Sustainability, Department of Chemistry, School of Natural and Computing Sciences, University of Aberdeen, Aberdeen AB24 3FX, U.K.

## Abstract

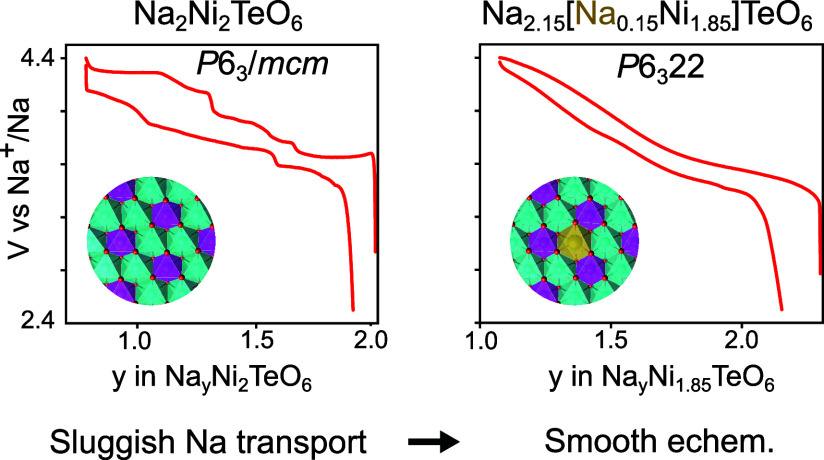

Layered Na_*x*_MO_2_ sodium oxide positive electrode materials have experienced renewed interest owing to the current commercial attention on sodium-ion batteries. Although there are many attractive qualities of these materials, they suffer from serious shortcomings owing to Na^+^ ordering and transition-metal layer gliding that cause a plethora of voltage plateaus during cycling. The P2-layered Na_2+*x*_Ni_2–*x*/2_TeO_6_ (0 ≤ *x* ≤ 0.5) system provides a framework for investigating the effect of dual Na^+^ substitution into the sodium layer and the transition-metal layer of the structure and its effects on the electrochemical properties of the materials. A careful investigation into the synthesis and properties of these materials reveals that the sodium content used during material preparation has a drastic effect on the composition and electrochemical profile of these materials. The sodium substitution disrupts ordering within the transition-metal layer, thereby disrupting Na^+^ ordering in the adjacent sodium layers. Beyond a critical sodium concentration, the layer stacking shifts, and all voltage plateaus of the P2-Na_2_Ni_2_TeO_6_ material are no longer observed at 4.4 V versus Na^+^/Na. These results also question the common belief that additional sodium precursor is required when preparing layered sodium oxide cathodes, providing new guidelines for material synthesis and characterization.

## Introduction

Although lithium batteries have emerged as the near-term solution for electrification of mobility and transitioning society toward a sustainable existence, there remain applications where secondary lithium batteries are not the most attractive energy storage solution. Secondary sodium batteries maintain an attractive value proposition for large-scale grid applications and portable electronics that prioritize low-cost over energy and power density. However, secondary sodium batteries have yet to achieve the critical performance metrics required to serve as a viable alternative for these applications. Layered Na_*x*_MO_2_ oxides remain the prime candidate for enabling a commercial Na-ion battery that has a reasonable energy density and rate performance, but most investigated materials suffer from sodium ordering and layered–layered phase transitions that induce numerous voltage plateaus in their electrochemical curve.^1,2^ The presence of numerous plateaus in the voltage curve for these materials upon sodium intercalation/deintercalation hinders their practicality.

Layered Na_*x*_MO_2_ oxides that present honeycomb ordering within the MO_2_ layer have developed increasing interest owing to their crystal and electronic structural nuances that play a role in seemingly superior electrochemical performance.^3,4^ These materials require a high valent ion, such as Sb^5+^ or Te^6+^, or a large size discrepancy between the intralayer metal ions to induce their honeycomb ordering. The fast Na^+^ ion conductivity of honeycomb ordered Na_*x*_MO_2_ oxides has also made them promising candidates as solid-state electrolytes.^5−7^ In a previous study, we studied the novel O’3-Na_3_Ni_1.5_TeO_6_ composition with the intent on substituting a small amount of Na^+^ for Ni^2+^ within the transition-metal layer of the material.^3^ This substitution resulted in a suppression of MO_2_ layer gliding during cycling that led to superior electrochemical performance of the material. The material demonstrated a singular phase transition from O’3 to P’3 once a small amount of sodium was extracted without the presence of sodium ordering in the interlayer space and a potentiostatic hold was required to reinsert enough Na^+^ to form the original O’3 structural type. The singular phase transition and lack of sodium ordering in the material during cycling resulted in a single sloping plateau voltage curve that is promising toward developing practical layered Na_*x*_MO_2_ cathode for sodium batteries. Additionally, the P’3-layered structure allowed for superior Na^+^ diffusion in the interlayer space leading to good rate capability.

From the results from the O’3-Na_3_Ni_1.5_TeO_6_ phase, the next apparent step became to assess whether a similar substitution strategy could be applied to a material that started with trigonal prismatic sites in the interlayer space to take advantage of the fast Na^+^ diffusion in the interlayer space from the start of cycling. A natural choice for this purpose was the P2–Na_2_Ni_2_TeO_6_ material of the same compositional family, which has previously been electrochemically evaluated by Gupta et al.^8^ After considering the charge balance of substituting Na^+^ for Ni^2+^ in this system, the final formula for evaluation is Na_2+*x*_Ni_2-*x*/2_TeO_6_ (or Na_2/3+*x*/6_[Na_*x*/6_Ni_2/3-*x*/6_Te_1/3_]O_2_). Of particular interest for this material was to observe how much sodium can be substituted while maintaining a purely P2 layer stacking as well as if substituting Na in the MO_2_ layer would induce a shift in the interlayer stacking. This system turns out to be extremely sensitive to the total sodium content in the material; the structure and the electrochemical properties are profoundly impacted by the pristine material composition. Elucidating the relationship between starting composition, structure, and electrochemical properties for these materials will guide further efforts of tailoring the performance of Na_*x*_MO_2_ layered oxide materials toward enabling a truly competitive low-cost rechargeable sodium battery.

## Experimental and Computational Methods

### Synthesis

The series of P2-layered Na_2+*x*_Ni_2-*x*/2_TeO_6_ (0 ≤ *x* ≤ 0.5) materials was prepared with traditional solid-state synthesis. Na_2_CO_3_ (Acros Organics, 99.8%), NiO (Alfa Aesar, 99%), and TeO_2_ (Alfa Aesar, 99.99%) were used as received without further purification as the starting materials. Stochiometric amounts of each precursor were ground in a mortar and pestle until a homogeneous powder was obtained. No excess sodium was used at any point in the synthesis of each composition and careful attention was paid to the Na/Ni ratio of the precursor materials. An initial firing of the powder was performed in an alumina boat at 650 °C for 12 h with a 10 °C per minute heating and cooling rate. This powder was then reground, pressed into a pellet, and fired at 810 °C for 24 h with a 10 °C per minute heating rate and 2 °C per minute cooling rate to obtain the final material. All furnace firings were performed in air.

### X-ray Diffraction and Refinement

All powder X-ray diffraction measurements were performed with a Rigaku Miniflex diffractometer (Cu Kα radiation). Powder diffraction patterns of all pure phases used for Le Bail refinement were obtained from 10 to 100° 2θ in stepping mode at 0.02° increments with a 2 s pause at each step. Powder diffraction patterns for phase verification that were not refined in any manner were obtained from 10° to 80° 2θ in continuous scanning mode at a scan rate of 5° per minute. Le Bail fitting of each pure phase material was performed with the FullProf software suite to obtain unit cell parameters and ensure proper identification of the space group for each material. All structural depictions in this work were developed with the VESTA 3D structure visualization program.

### Solid-State Nuclear Magnetic Resonance Spectroscopy

Hahn echo ^23^Na magic angle spinning (MAS) nuclear magnetic resonance (NMR) spectra were recorded with a Bruker 300 Avance spectrometer at 79.47 MHz at spinning frequencies of 28 kHz and 30 kHz. Each material was loaded into a zirconia rotor in an Ar-filled glovebox before being loaded into the spectrometer for measurement. A Hahn-echo pulse sequence with a pulse length of 2 μs and a recycle time of D1 = 0.2 s was used for spectrum acquisition. The external reference was a 0.1 M NaCl aqueous solution.

^23^Na solid-state NMR spectra were also acquired with the pj-MATPASS pulse sequence on a 400 MHz Bruker Avance III HD spectrometer to further support the assignment of different ^23^Na resonances.^9^ A Bruker 4 mm HXY probe was used for pj-MATPASS spectra at a MAS frequency of 8 kHz. 64 slices in the F2 dimension and 640 scans per slice were used in the pj-MATPASS pulse sequence. A π/2 pulse length of 2.2 μs, optimized on solid NaCl at a power of 80 W, was used for all pj-MATPASS measurements. NaCl was used as a secondary shift reference at 7.21 ppm (relative to 1 M NaCl(aq)). pj-MATPASS spectra were obtained with a short recycle delay of 0.01 or 0.1 s to selectively enhance fast relaxing paramagnetic environments.

### Electrode Preparation

Electrodes for galvanostatic cycling were prepared with 70 wt % active material, 20 wt % Denka black as the electronically conductive additive, and 10 wt % polytetrafluoroethylene (PTFE) as the binder. For each electrode, the active material and Denka black were thoroughly ground together in a mortar and pestle before adding in the PTFE to the mixture. The mixture was then ground until a homogeneous film was obtained and rolled into a free-standing electrode film. This film was dried at 80 °C for at least 12 h before electrode discs 1/4 in. in diameter were punched, weighed, and transferred into an Ar-filled glovebox (MBraun) with H_2_O and O_2_ levels below 0.1 ppm for electrochemical cell assembly.

### Coin Cell Assembly and Electrochemical Testing

2032-coin cells were fabricated with a P2-layered Na_2+*x*_Ni_2-*x*/2_TeO_6_ (0 ≤ *x* ≤ 0.5) electrode composite disc as the cathode, glass-fiber (Whatman) as the separator, and a sodium metal anode. 1 M NaClO_4_ in propylene carbonate (PC): fluoroethylene carbonate (FEC) in a volume ratio of 9:1 was used as the electrolyte for these cells. Galvanostatic cycling measurements were performed with LANHE battery testing units. All electrochemical cycling of the P2-layered Na_2+*x*_Ni_2-*x*/2_TeO_6_ (0 ≤ *x* ≤ 0.5) materials in this study were performed at a C/20 cycling rate with different voltage cutoffs between 2.5 ≤ V ≤ 4.4 V versus Na^+^/Na.

### Computational Methods

Spin polarized density functional theory (DFT) calculations were performed with the projector augmented wave (PAW) approach in the VASP code.^10,11^ The Perdew–Burke–Ernzerhof (PBE) functional was used for all calculations and a Hubbard U parameter (DFT+U) was applied to Ni to correct for issues associated with electron localization.^12^ The rotationally invariant form of DFT+U proposed by Dudarev et al. was used with a U value of U_eff_ = 6.2 eV for Ni, based on previous work.^13,14^

Low energy Na orderings in stoichiometric and Na substituted Na_2+*x*_Ni_2-*x*/2_TeO_6_ structures were predicted via a Monte Carlo (MC) basin hopping approach inspired by Wales and Doye.^15^ The energy of sodium orderings were taken from DFT calculations from VASP. This approach was previously used to find low energy Li orderings in the analogous Li_2_Ni_2_TeO_6_ system.^16^ For the pristine and Na-substituted *P*6_3_/*mcm* and *P*6_3_22 phases of Na_2+*x*_Ni_2-*x*/2_TeO_6_, 88–90 atom supercells were created with a random distribution of Na on the Na1, Na2 and Na3 (Na3′) sites. In each MC step, the position of possible Na vacancies was located by finding Voronoi polyhedral with the Zeo++ code, as implemented in the pymatgen package.^17,18^ To ensure sufficient space for Na atoms to be inserted into the vacancy, only Voronoi polyhedra greater than 2 Å away from a neighboring atom were considered. The position of an occupied Na site and a vacant site were then randomly selected and swapped. After each swap, depending on the MC temperature, either the atomic positions (high temperature: 1000 and 2000 K) or the atomic positions and the cell parameters (low temperature: 300 K) were fully optimized until the force on any atom fell below 0.1 eV Å^–1^. The final energy of the cell after optimization was used as the trial energy in the Metropolis Monte Carlo selection step. For calculations run at successive temperatures, the atomic positions and the lattice parameters of the lowest energy structure found in each range were fully optimized and used as the input for the next temperature range. A dispersion correction (DFT+D3) based on the zero damping Grimme method was added to VASP calculations, unless otherwise stated, to improve the description of van der Waals interactions between the layers.^19^

Coarse DFT settings were used for the basin hopping procedure to minimize the computational cost. The planewave cut off energy was set to 400 eV, with an energy criterion of 10^–4^ eV. The softest pseudopotentials available in VASP were used for each element (Na, Ni, Te and O_s). The Brillouin zone was sampled with a single k-point at the gamma point. Tighter energy convergence (10^–6^ eV) and plane wave cut off energies (600 eV) were used for the energy evaluation of the final, lowest energy structures at 300 K. A gamma-centered k-point mesh density of 28 Å was used for the final energy evaluations.

Calculations were also performed in this work with the MACE-MP-0 machine learning force field (ML-FF) to study the energetics and dynamics of larger Na_2+*x*_Ni_2-*x*/2_TeO_6_ supercells.^20^ The MACE-MP-0 ML-FF has been shown to give a good approximation for the DFT energies of battery systems including transport at the electrode/electrolyte interface.^20^ Using forces and energies from MACE-MP-0, MC basin hopping calculations were performed on Na_2.25_Ni_1.875_TeO_6_ supercells with the *P*6_3_/*mcm* and *P*6_3_22 structures. 100 MC swapping steps were performed at 1000 K under fixed volume conditions, followed by 200 steps at 300 K in which the atoms and cell parameters were allowed to vary at each step. The lowest energy configuration predicted for each structure was then fully optimized with DFT using the tighter energy convergence criteria described previously.

Molecular dynamics (MD) simulations were also performed with the MACE-MP-0 potential on Na_2+*x*_Ni_2-*x*/2_TeO_6_ supercells containing 16 formula units, to explore the impact of Na composition (*x*) and layer stacking on Na dynamics. MD simulations were performed with the Atomic Simulation Environment (ASE) package in the NVT ensemble with a Berendsen thermostat at 500 K.^21,22^ Simulations were first equilibrated for 20 ps, followed by a production run of 100 ps. A time step of 2 fs was used for all simulations. The self-diffusion coefficient (*D*_Na_) of Na was calculated from the mean-square displacement (MSD) ⟨[*r*(*t*)]^2^⟩ against time (*t*) via the equation:
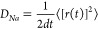
Where *d* is the dimensionality of conduction, taken here to be 3.

^23^Na NMR Fermi contact shifts of the low energy Na_2_Ni_2_TeO_6_ and Na_2.5_Ni_1.75_TeO_6_ structures predicted by MC swapping were calculated with nominally 0 K DFT calculations in VASP with DFT+U, based on the method outlined in previous studies.^23−26^ Although hybrid functionals have previously been shown to improve the description of Fermi contact interactions compared to DFT+U methods, quantitative agreement between DFT+U calculations and experimental NMR spectra was previously found for a related system, Na_3_Ni_2_SbO_6_.^4,27^

Fermi contact shift contributions were scaled from 0 K to finite temperature via a scaling factor, Φ:
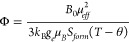
where *B*_*0*_ is the external magnetic field (9.4 T), *k*_*B*_ is Boltzmann’s constant, *S*_*form*_ is the formal spin angular momentum quantum number of Ni^2+^ (*S*_*form*_ = 1), *g*_*e*_ is the free electron g factor (equal to 2.0023), μ_*B*_ is the Bohr magneton, *T* is the experimental temperature, μ_*eff*_ is the effective magnetic moment and θ is the Weiss constant.^24^

The value of *T* at 30 kHz MAS was taken as 320 K to account for frictional heating. The spin only value of μ_*eff*_ = 2.83 μ_*B*_ was used for Ni^2+^. A Weiss constant of θ = −32 K taken from previous magnetic characterization of Na_2_Ni_2_TeO_6_.^28^ For Fermi contact calculations, pseudopotentials containing additional valence electrons were used for several elements (Na_pv, Ni_pv and O) to improve the description of the electronic structure. The plane wave energy cut off and energy convergence criteria were also increased for Fermi contact calculations from 600 eV and 10^–7^ eV, respectively.

## Results and Discussion

### Synthesis and Structure

The phase purity of the series of P2-layered Na_2+*x*_Ni_2-*x*/2_TeO_6_ (0 ≤ *x* ≤ 0.5) materials was assessed with powder X-ray diffraction. Figure S1 (Supporting Information) shows the resulting X-ray diffraction patterns of each material and provides the classical layered formula for each composition with the assumption that sodium occupies the vacancies in the MO_2_ layer created by the nickel deficiency during synthesis — the validity of this hypothesis will be discussed further below; however, the point at which a secondary layered phase emerges in the compositional series is a preliminary indication that this hypothesis is possible. Compositions beyond Na_2.3_Ni_1.85_TeO_6_ show the emergence of an additional layered phase other than the desired P2-layered structure. The secondary phase in the Na_2.4_Ni_1.8_TeO_6_ and Na_2.5_Ni_1.75_TeO_6_ compositions has lower *d*-spacing of the (00*l*) peak, which can be attributed to an O3-stacking layered phase. In previous work by the current authors, a single O’3 phase was found to be present at the composition Na_3_Ni_1.5_TeO_6_ (Na_5/6_[Na_1/6_Ni_3/6_Te_2/6_]O_2_).^3^ The preference for O3 stacking over P2 stacking in other as-synthesized Na_*x*_MO_2_ systems has also been observed when the Na^+^ composition in the Na layer exceeds Na_0.7_MO_2_.^1,29^ The exact composition of secondary O3 phase in the current study is unknown, but the result highlights the limit of Na^+^ substitution for Ni^2+^ in the P2 Na_2+x_Ni_2-x/2_TeO_6_ system. For the remainder of the study, structural characterization was only performed on the P2 materials in the compositional range of 0 ≤ *x* ≤ 0.3.

**Figure 1:**
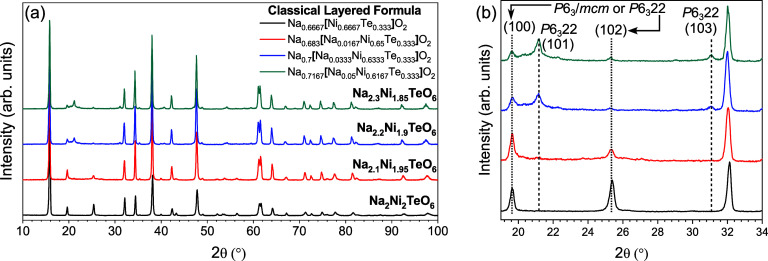
a) Powder X-ray diffraction patterns for pure phase Na_2+*x*_Ni_2-*x*/2_TeO_6_ (0 ≤ *x* ≤ 0.3) materials with a zoom in of the superstructure region of 19° to 34° 2θ shown in b).

Figure 1a shows the X-ray diffraction patterns of only the pure phase Na_2+*x*_Ni_2-*x*/2_TeO_6_ (0 ≤ *x* ≤ 0.3) materials with Figure 1b providing an enhanced view of the superstructure region of the diffraction patterns. The major peaks in the Na_2_Ni_2_TeO_6_ diffraction pattern can be indexed to the *P*6_3_/*mcm* space group. However, where x = 0.1 for the Na_2.1_Ni_1.95_TeO_6_ composition, an extra peak begins to emerge at 2θ ≈ 21.2° that is forbidden in the *P*6_3_/*mcm* space group. This peak, along with a second peak at 2θ ≈ 31° that is also forbidden in the *P*6_3_/*mcm* space group, continue to increase in intensity with increasing sodium concentration in the as-synthesized materials. In the original report of the Na_2_M_2_TeO_6_ (M = Ni, Co, Mg, or Zn) family of materials, only Na_2_Ni_2_TeO_6_ demonstrated the *P*6_3_/*mcm* space group for its P2-layered structure; the other three materials demonstrated a P2-layered structure with the space group *P*6_3_22.^7^ Additionally, the Na_2_Ni_2_TeO_6_ material changed from the *P*6_3_/*mcm* space group to the *P*6_3_22 with the introduction of a small amount of lithium substitution for nickel in the MO_2_ layer for the composition Na_2.1_Ni_1.9_Li_0.1_TeO_6_. From this observation, the X-ray diffraction patterns of Na_2+*x*_Ni_2-*x*/2_TeO_6_ (0.2 ≤ *x* ≤ 0.3) were fully indexed to the *P*6_3_22 space group with all peaks being accounted for.

The preliminary observations from the superstructure region of the diffraction patterns were assessed and confirmed by performing a Le Bail refinement on each X-ray diffraction pattern for Na_2+*x*_Ni_2-*x*/2_TeO_6_ (0 ≤ *x* ≤ 0.3). The results of the Le Bail refinements are provided in Figure S2–S5 with a summary of the lattice parameters and reliability factors for each refinement given in Table 1. For the Na_2.1_Ni_1.95_TeO_6_ composition, it was necessary to consider two phases with space groups *P*6_3_/*mcm* and *P*6_3_22 to obtain acceptable reliability factors for the refinement with the *P*6_3_/*mcm* being the dominant contribution to the diffraction pattern. The reliability factors notably drop off for the Na_2.2_Ni_1.9_TeO_6_ and Na_2.3_Ni_1.85_TeO_6_ compositions and the calculated diffraction patterns deviate from the experimentally observed diffraction pattern a bit more than desired (see Figures S4 and S5, Supporting Information), particularly in the superstructure region. This deviation is attributed to the presence of stacking faults in the material. Stacking faults are known to cause anisotropic peak broadening that can prevent a good Le Bail fit.^30,31^ The broadening of the (101) and (103) peaks as a function of stacking fault percentage has been studied previously in the isostructural Na_2_Zn_2_TeO_6_ compound.^32^ The broadening of the (101) and (103) peaks in the current Na_2.2_Ni_1.9_TeO_6_ and Na_2.3_Ni_1.85_TeO_6_ materials resembles the pattern of Na_2_Zn_2_TeO_6_ with a stacking fault percentage of < 10%.^32^ Broadening of other peaks such as the (103), (115), (122), (205) and (123) peaks is also observed (Figure S6a) which is consistent with stacking faults between the *P*6_3_22 and *P*6_3_/*mcm* structures. In the case of Na_2.2_Ni_1.9_TeO_6_ and Na_2.3_Ni_1.85_TeO_6_, selective peak broadening due to stacking faults causes reliability factors for the Le Bail fits of these two materials to be higher than in the case of a layered structure with perfect stacking. Nevertheless, the lattice parameters determined from the peak positions from these refinements provide insight into the structural depiction of these materials.

**Table 1:** Unit Cell Information Obtained from Le Bail Fitting the Powder X-ray Diffraction Patterns of Na_2+*x*_Ni_2-*x*/2_TeO_6_ (0 ≤ *x* ≤ 0.3)

Composition	*x* = 0	*x* = 0.1		*x* = 0.2	*x* = 0.3
Space group	*P*6_3_/*mcm*	*P*6_3_/*mcm*	*P*6_3_22	*P*6_3_22	*P*6_3_22
lattice parameters (Å)					
a = b	5.2038 (2)	5.2084 (1)	5.1756 (34)	5.2218 (2)	5.2278 (1)
c	11.1399 (5)	11.1544 (3)	11.2461 (38)	11.1932 (5)	11.1829 (4)
reliability factors					
R_wp_	10.8	11.9		18.1	17.4
R_Bragg_	1.12	1.5		2.57	3.0
χ^2^	5.16	3.98		9.78	8.73

On close inspection of the region from 26–31° 2θ, two very weak peaks at 28.9 and 30.0° are visible in the Na_2_Ni_2_TeO_6_ phase (Figure S6b). Peaks in this region are commonly assigned to the presence of Na ordering, such as the “large zigzag” ordering, which has been proposed in P2–Na_*x*_CoO_2_ and Na_0.67_Ni_1/3_Mn_2/3_O_2_ materials.^33,34^ Further refinement of these peaks is not possible from lab-based X-ray diffraction, but the result highlights that Na^+^ ordering is present in the Na_2_Ni_2_TeO_6_ end member. On increasing the Na content to Na_2.1_Ni_1.95_TeO_6_ the Na^+^ ordering peaks disappear. At compositions of Na_2.2_Ni_1.9_TeO_6_ and Na_2.3_Ni_1.85_TeO_6_, a very weak additional peak is observed at 29.0° 2θ, which is not present in the *P*6_3_22 space group. Future studies with high energy synchrotron radiation are required to assign this peak to either Na^+^/vacancy ordering or a small amount of impurity phase.

The c-lattice parameter evolution indicates which sites the additional sodium is occupying when substituted for nickel. If all the sodium were occupying the interlayer space without any sodium in the MO_2_ layer to fill the Ni vacancies, then the c-lattice parameter would be expected to continuously decrease with increasing sodium concentration owing to the greater cohesive force between adjacent MO_2_ layers. However, from the Na_2_Ni_2_TeO_6_ to the Na_2.1_Ni_1.95_TeO_6_ composition, the c-lattice parameter increases for both stacking sequences present in the latter material. Thus, there is evidence that sodium is occupying the vacant nickel sites in the MO_2_ layer. For the Na_2.2_Ni_1.9_TeO_6_ and Ni_2.3_Ni_1.85_TeO_6_ materials, as the sodium concentration in the interlayer space increases, the c-lattice parameter decreases, but is still larger than the c-lattice parameter of the initial Na_2_Ni_2_TeO_6_ material. Therefore, beyond the Na_2.1_Ni_1.95_TeO_6_ composition, the contraction of the c-lattice parameter induced by cohesive force of the additional Na^+^ in the interlayer space dominates the expansion caused by the sodium substitution of Ni in the MO_2_ layer.

**Figure 2:**
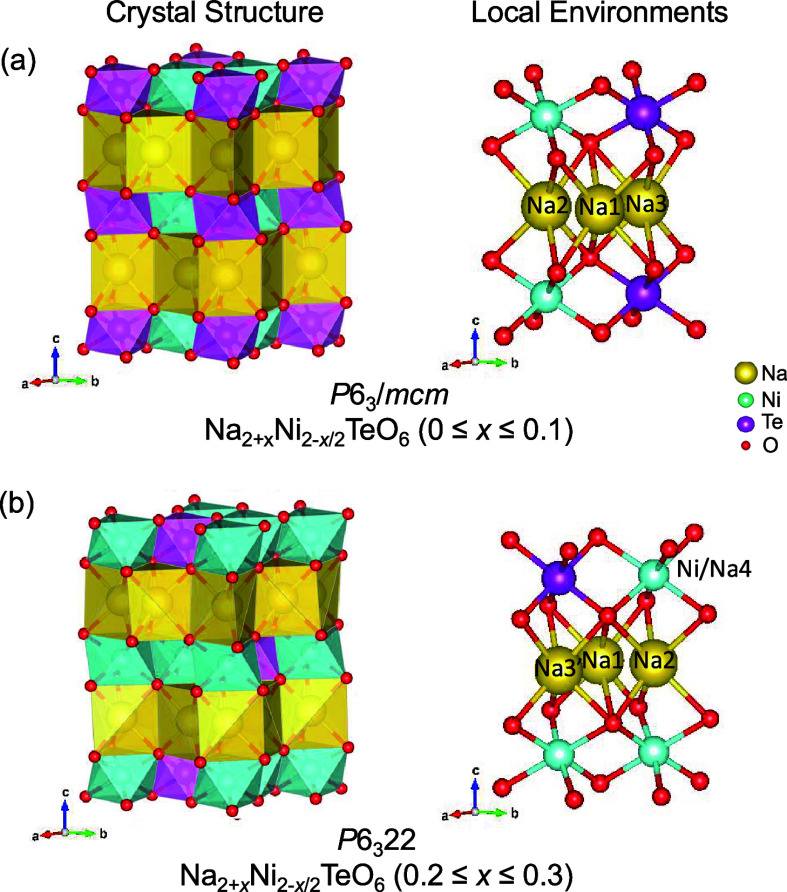
Depiction of the crystal structures and local sodium environments for the *P*6_3_/*mcm* and *P*6_3_22 P2-layered structures observed in the Na_2+*x*_Ni_2-*x*/2_TeO_6_ (0 ≤ *x* ≤ 0.3) system. Na1, Na2, Na3, Na3′, and Ni/Na4 are used to denote the different sodium environments in each structure. Na1 is face sharing with vacant tetrahedral sites within the adjacent MO_2_ layers. Na2 is face-sharing with only NiO_6_ octahedra in the MO_2_ layers. Na3 is face-sharing with only TeO_6_ octahedra in the MO_2_ layers. Na3′ is face-sharing with an NiO_6_ octahedra in one MO_2_ layer and a TeO_6_ octahedra in the other MO_2_ layer. Ni/Na4 are partial Ni^2+^ and Na^+^ occupancy sites within the MO_2_ layer.

With the Le Bail refinements confirming the indexed space groups, structural models could be developed by comparison to the original report of Na_2_M_2_TeO_6_.^7^ These structural models are shown in Figure 2 along with the relevant sodium environments within each structure. Although both structures fall under the classification of P2-layered structures according to Delmas notation, there are notable differences in their MO_2_ stacking, which account for the difference in superstructure.^35^ Each structure demonstrates an in-plane honeycomb ordering within the MO_2_ layer where every TeO_6_ octahedra is surrounded by six [Na/Ni]O_6_ octahedra. These honeycomb ordered [Na_*x*/6_Ni_2/3-*x*/6_Te_1/3_]O_2_ layers can then be stacked in different configurations to form the *P*6_3_/*mcm* and *P*6_3_22 space group structures for this system. In the P2-layered structure with a *P*6_3_/*mcm* space group (Figure 2a), each MO_2_ layer is rotated 180° from the MO_2_ layer above it. In this structure, each NiO_6_ octahedra is oriented directly above another NiO_6_ octahedra and each TeO_6_ octahedra is oriented directly above another TeO_6_ octahedra. For the *P*6_3_22 space group, (Figure 2b) the second MO_2_ layer in the unit cell is rotated clockwise 90° from the MO_2_ layer above it. This MO_2_ layer stacking sequence creates multiple different sodium environments in the interlayer space, since the TeO_6_ octahedra are not oriented directly above another TeO_6_ in the adjacent MO_2_ layer. The *P*6_3_/*mcm* P2-layered structure has three trigonal prismatic Na^+^ sites within the interlayer space: one site that is face sharing with vacant tetrahedral sites within the adjacent MO_2_ layers (Na1), one site that is face-sharing with only NiO_6_ octahedra in the MO_2_ layers (Na2), and one site that is face-sharing with only TeO_6_ octahedra in the MO_2_ layers (Na3). The *P*6_3_22 P2-layered structure also has three sodium sites in the interlayers space: one site that is face sharing with empty tetrahedral sites in the MO_2_ layers (Na1), one site that is only face-sharing with (Na/Ni)O_6_ octahedra (Na2), and one site that is face-sharing with an NiO_6_ octahedra in one MO_2_ layer and a TeO_6_ octahedra in the other (Na3′). However, this structure has a potential fourth Na^+^ site that resides within the MO_2_ layer if the sodium replaces Ni^2+^ in the layer (Na4), as the compositions suggest.

Upon inspection of the two different P2-layered stacking configuration, electrostatic arguments would indicate the MO_2_ stacking of the *P*6_3_22 space group should cause less sodium ordering in the sodium layer since the Coulombic interactions of the Te^6+^ ions are not localized directly above one another. Thus, Na^+^ in the *P*6_3_/*mcm* structure would preferentially occupy trigonal prismatic sites within the sodium layer that are not face-sharing with two TeO_6_ octahedra in the MO_2_ layer above and below it–i.e. low Na3 site occupancy.

### Computational Investigation of Phase Stability

**Figure 3:**
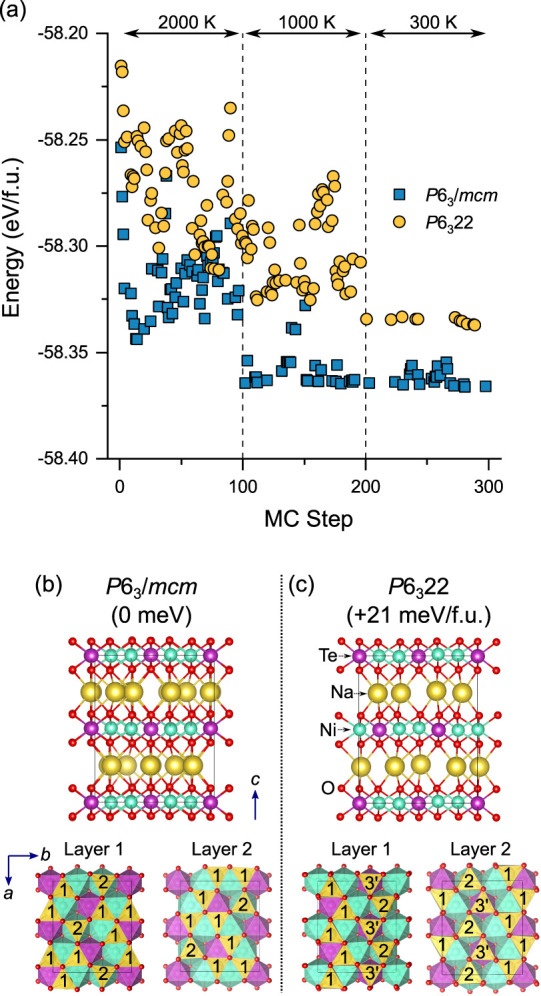
(a) DFT energetics of stoichiometric *P*6_3_/*mcm* and *P*6_3_22 structures with Monte Carlo basin hopping Na/V_Na_ swapping step. Only the energies of “accepted” structures are shown. At each MC step, the positions of all atoms were optimized under fixed cell conditions. Dashed lines indicate different temperature regions. After 100 MC steps at 2000 and 1000 K, the lowest energy structure found was fully optimized and used for the subsequent temperature step. (b) and (c) optimized supercell structures of lowest energy *P*6_3_/*mcm* and *P*6_3_22 structures, respectively, found at 300 K with MC. Relative energy difference between the structures is shown in meV per formula unit. The ordering of the Na1, Na2, and Na3′ (*P*6_3_22) sites in the two Na layers (ab plane) of both structures is shown.

The relative energies of the pristine *P*6_3_/*mcm* and *P*6_3_22 structures were studied with DFT calculations of Na_2_Ni_2_TeO_6_ supercells containing 8 formula units (Na_16_Ni_16_Te_8_O_48_). Possible low energy Na/Na vacancy (V_Na_) orderings at room temperature in both phases were investigated via Monte Carlo swapping in which the position of Na vacancies was located with Voronoi polyhedra. For the pristine Na_2_Ni_2_TeO_6_ structures, a multistep procedure was used in which 100 Na/V_Na_ swaps were carried out sequentially at 2000 K, 1000 K and finally, 300 K. The results for the pristine *P*6_3_/*mcm* and *P*6_3_22 Na_2_Ni_2_TeO_6_ structures are shown in Figure 3.

For the pristine Na_2_Ni_2_TeO_6_ structure, at 300 K, the energies of the *P*6_3_/*mcm* and *P*6_3_22 structures from the MC swapping process reached a plateau (Figure 3a), with the *P*6_3_/*mcm* structure resulting in a lower in energy than the *P*6_3_22 structure. The lowest energy configurations found for both structures were selected and fully optimized to a tighter tolerance with DFT. The lowest energy *P*6_3_22 structure was found to be 21 meV/formula unit higher in energy than the lowest energy *P*6_3_/*mcm* structure (Figure 3b), which is in good agreement with the experimental XRD result in which the *P*6_3_/*mcm* structure is observed for pristine Na_2_Ni_2_TeO_6_ at room temperature, in the absence of Na substitution.

The lowest energy *P*6_3_/*mcm* structure contained a 3:1 ratio of Na1 to Na2 sites (Figure 3b), while the ratio of Na1, Na2 and Na3′ sites in the lowest energy *P*6_3_22 structure was 2:1:1. The Na1 trigonal prismatic sites are low energy positions in both structures, which share edges with 4 NiO_6_ and 2 TeO_6_ octahedra, minimizing their electrostatic repulsion. The Na2 sites in both structures share a common face with two NiO_6_ sites. The Na3 sites, which share faces with two TeO_6_ octahedra, were not present in the lowest energy *P*6_3_/*mcm* structure. This result is consistent with the high electrostatic repulsion between Na^+^ and Te^6+^ cations in these configurations. For the *P*6_3_22 structure, the Na were present in Na3′ sites, which are face sharing with one NiO_6_ in the layer above (below) and one TeO_6_ in the layer below (above). The electrostatic repulsion of these sites is therefore expected to be intermediate between the Na2 (2 face sharing NiO_6_) and Na3 (2 face sharing TeO_6_) sites in the P6_3_/*mcm* structure.

**Figure 4:**
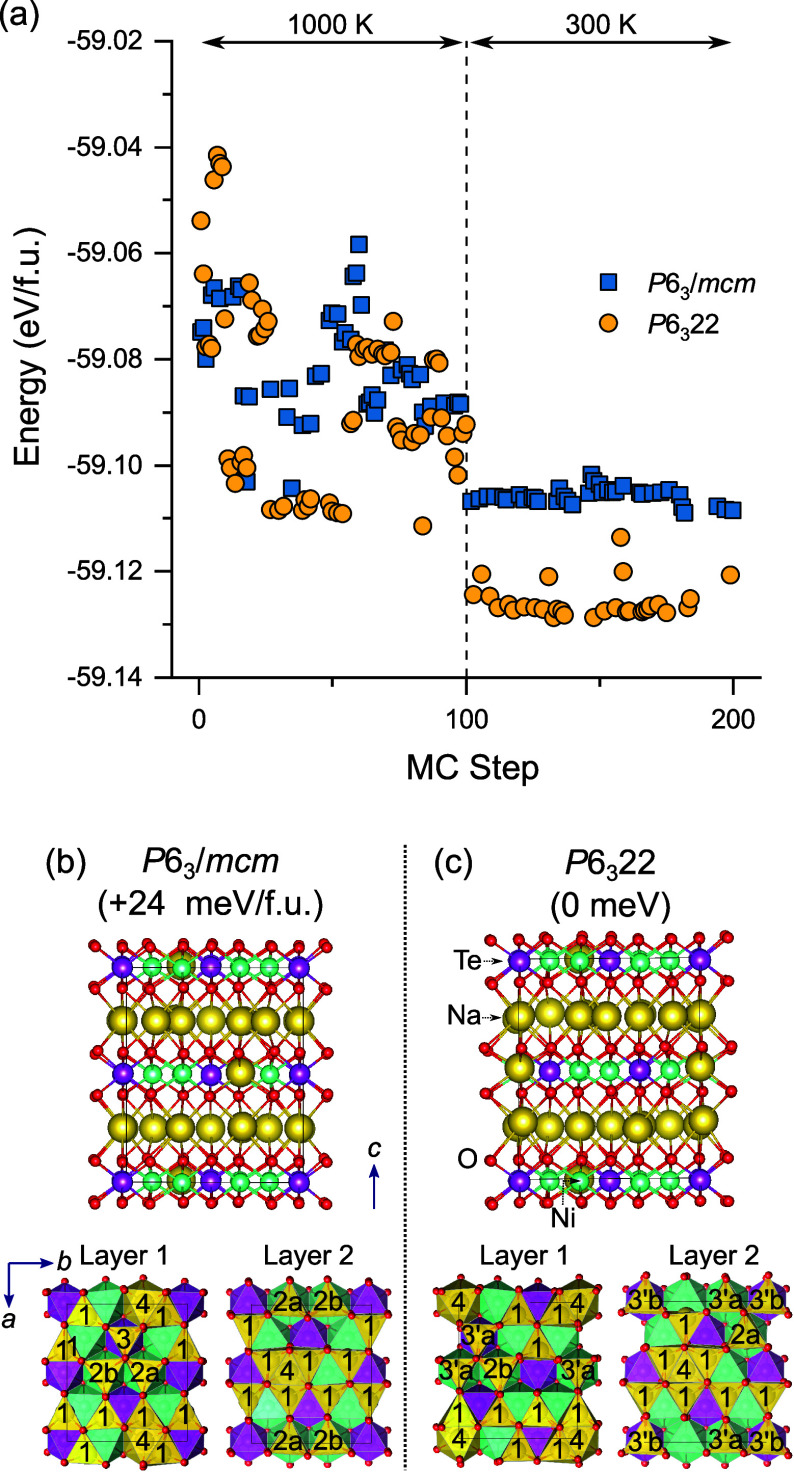
(a) DFT energetics of Na^+^ substituted *P*6_3_/*mcm* and *P*6_3_22 structures with Monte Carlo basin hopping Na/V_Na_ swapping step. Only the energies of “accepted” structures are shown. At each MC step, the positions of all atoms were optimized under fixed cell conditions. The dashed line indicates the different temperature regions. After 100 MC steps at 1000 K, the lowest energy structure found was fully optimized and used for the subsequent temperature step. (b) and (c) optimized supercell structures of lowest energy *P*6_3_/*mcm* and *P*6_3_22 structures, respectively, found at 300 K with MC. Relative energy difference between the structures is shown in meV per formula unit. The ordering of the Na1, Na2 and Na3′ (*P*6_3_22) sites in the two Na layers (ab plane), and Na4 site in the Ni–Te layer of both structures is shown.

The impact of Na^+^ substitution into the Ni^2+^ sites for Na-excess materials with the *P*6_3_/*mcm* and *P*6_3_22 structures was further studied with the MC basin hopping approach. Two Ni^2+^ sites in the lowest energy *P*6_3_/*mcm* and *P*6_3_22 pristine structures were substituted for Na^+^, one in each layer. Two additional Na^+^ ions were then randomly added to vacant sites in both structures for charge balance to create a Na-excess Na_2.5_Ni_1.75_TeO_6_ (Na_18_[Na_2_Ni_14_Te_8_]O_48_) composition. 100 MC swapping steps were performed at 1000 K followed by 100 MC steps at 300 K. Analogous to the pristine material, the lowest energy structure from the first 100 MC steps at 1000 K was fully optimized and used as the input for the 300 K runs. The results for the Na substituted *P*6_3_/*mcm* and *P*6_3_22 structures is shown in Figure 4a.

At 300 K, the *P*6_3_22 structure of Na_2.5_Ni_1.75_TeO_6_ was found to be lower in energy than the *P*6_3_/*mcm* structure (Figure 4a) from MC swapping, opposite to what was previously found for the pristine Na_2_Ni_2_TeO_6_ phase (Figure 3a). The lowest energy *P*6_3_/*mcm* and *P*6_3_22 phases were fully optimized with tighter DFT settings, and the former phase was found to be 24 meV/formula unit higher than the latter phase (Figure 4b). The change in the phase stability from the *P*6_3_/*mcm* to the *P*6_3_22 structure is in excellent agreement with experiment, where Na substitution was found to progressively lead to the *P*6_3_22 phase.

There is a tendency for the Na^+^ sites in the Ni/Te layer to be coordinated by Na1 sites in the adjacent Na layers, from analysis of the Na^+^ positions in the lowest energy Na substituted *P*6_3_/*mcm* and *P*6_3_22 structures (Figure 4b). This ordering creates islands of Na1 sites that help disrupt the long-range Na ordering. The presence of Na^+^ in the Ni–Te layer (Na4) also alters the local environment of some of the face sharing Na2 and Na3′(*P*6_3_22) sites. To distinguish between when one of the Ni sites in the NiO_6_–Na-NiO_6_ or NiO_6_–Na-TeO_6_ face sharing configurations is substituted with Na, it is given the symbol ‘b’ (Na2b or Na3′b), whereas the original unsubstituted site is designated as ‘a’ (Na2a or Na3′a) in Figure 4b. The lower electrostatic repulsion between face sharing Na^+^-Na^+^ configurations compared to Na^+^-Ni^2+^ configurations is expected to further stabilize these sites. For the lowest energy *P*6_3_22 structure, all types of sites are present (Na1, Na2a, Na2b, Na3′a and Na3′b) with a preference for Na1 sites. For the *P*6_3_/*mcm* structure, all types of sites (Na1, Na2a, Na2b and Na3) are also observed, including the Na3 site. The Na3 TeO_6_–Na-TeO_6_ configuration is not altered by Na^+^ substitution on the Ni^2+^ site and is expected to remain as a high energy site. The preferential lowering in energy of the Na3′b sites in the *P*6_3_22 structure may further help to disrupt Na ordering, leading to fast Na^+^-ion diffusion.

The energies of the *P*6_3_/*mcm* Na_2_Ni_2_TeO_6_ and *P*6_3_22 Na_2.5_Ni_1.75_TeO_6_ phases were also calculated with DFT settings consistent with the Materials Project database.^36^ Both phases were found to be stable relative to known phases within the Na–Ni–Te-O phase diagram (Na_4_TeO_5_, Na_2_TeO_4_ and NiO). It should be highlighted that experimentally at a composition of *x* > 0.3, and additional O3-layered phase starts to form (Figure S1). The composition of this phase is unknown, although the O'3–Na_3_Ni_1.5_TeO_6_ phase is known to be stable experimentally.^3^ A low energy DFT structure has yet to be proposed for O'3–Na_3_Ni_1.5_TeO_6_, which contains both Na^+^/Ni^2+^ and Na^+^/vacancy disorder. Understanding the relative energies of layered O3 and P2 Na_2+x_Ni_2-x/2_TeO_6_ will be the focus of future studies with the methods developed in this work.

To further explore the relative stability of the *P*6_3_/*mcm* and *P*6_3_22 phases for a composition of 0 < *x* < 0.3, large Na_2.25_Ni_1.85_TeO_6_ (Na_36_Ni_30_Te_16_O_96_) supercells were created from a √2*a* × √2*b* expansion of the lowest energy Na_2.5_Ni_1.75_TeO_6_ structures. An Na4 site in each Ni–Te layer was swapped with Ni and an Na site was removed at random in each Na layer to give the correct stoichiometry. Due to the large size of the required supercells, extensive MC swapping calculations with explicit DFT energetics were not feasible. Machine learning force fields (ML-FF) based on the MACE-MP-0 potential were therefore adopted to give a good approximation of the energetics in the MC swapping procedure (Figure S7a). 100 MC swapping steps were performed for each structure at 1000 K in which the atomic positions were optimized in every step, followed by 200 MC swapping steps at 300 K in which the atomic positions and cell parameters were optimized in every step.

The lowest energy Na^+^ orderings predicted from the MC swapping procedure with ML-FF were then optimized with tight DFT (DFT+U+D3) convergence criteria, analogous to the Na_2_Ni_2_TeO_6_ and Na_2.5_Ni_1.75_TeO_6_ structures. The energy of the DFT optimized *P*6_3_22 Na_2.25_Ni_1.875_TeO_6_ structure (Figure S7b) was found to 25 meV/f.u. lower in energy than the *P*6_3_/*mcm* structure (Figure S7c), further highlighting that Na-excess stabilizes the *P*6_3_22 phase. The Na_2.25_Ni_1.875_TeO_6_ structure found by the combined ML-FF + DFT approach has a decomposition energy of only 13 meV/f.u. (1 meV/atom) relative to the lowest energy *P*6_3_/*mcm* Na_2_Ni_2_TeO_6_ and *P*6_3_22 Na_2.5_Ni_1.75_TeO_6_ structures found through purely DFT MC swapping. This result highlights that the weakly metastable Na_2.25_Ni_1.875_TeO_6_ phase may be stabilized entropically at synthesis temperatures. The results also highlight that ML-FF coupled with MC basing hopping approaches are a powerful tool for exploring the phase space of novel Na^+^/vacancy ordered cathode materials.

### Molecular Dynamics Simulations

**Figure 5:**
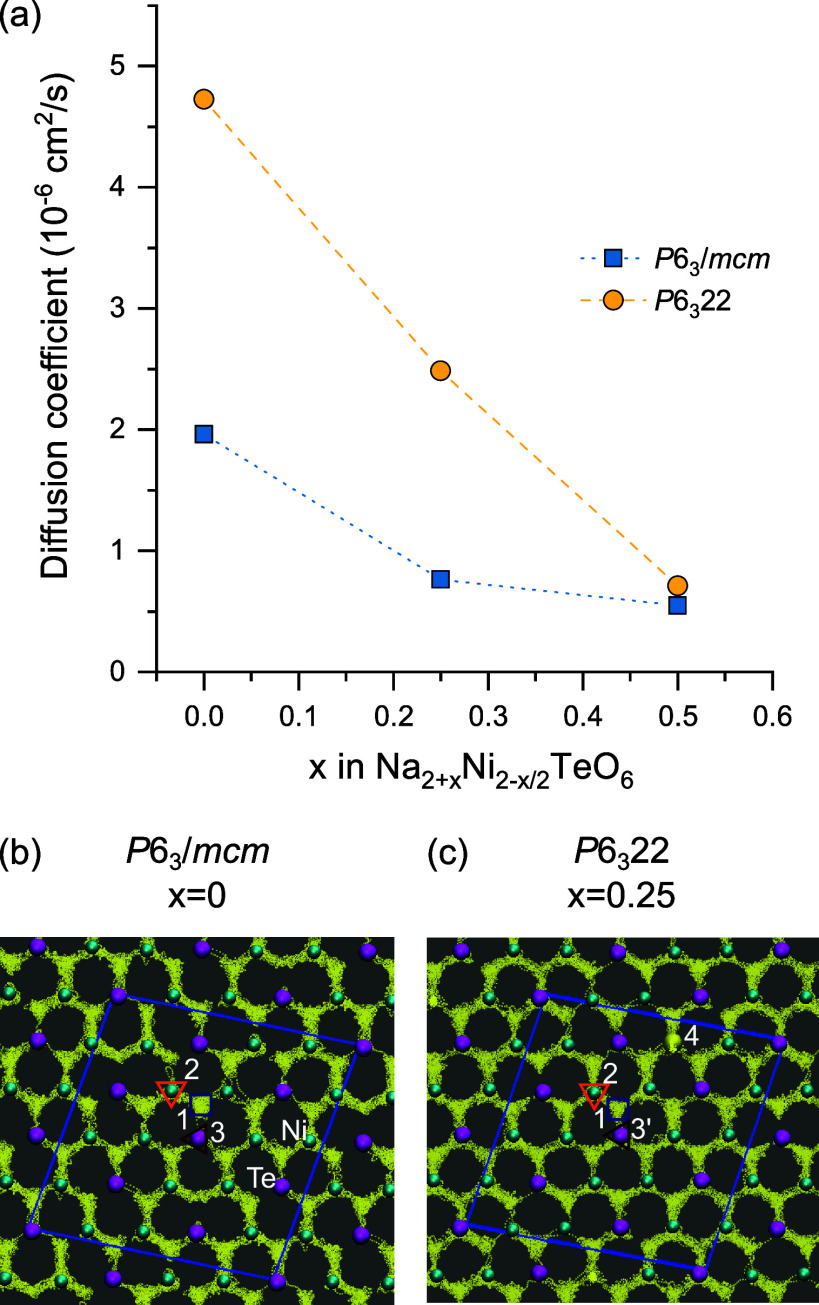
(a) Plot of Na diffusion coefficient at different *x* compositions in the *P*6_3_/*mcm* and *P*6_3_22 Na_2+*x*_Ni_2-*x*/2_TeO_6_ structures from molecular dynamics simulations at 500 K with the MACE-MP-0 potential. Trajectories of all Na atoms over the 100 ps MD run within the c = 0.75 layer of the (b) *P*6_3_/*mcm* Na_2_Ni_2_TeO_6_ and (c) *P*6_3_22 Na_2.25_Ni_1.875_TeO_6_ structures are shown as yellow dots. The positions of the Na1, Na2, Na3/Na3′ sites for both structures are shown as a blue square, a downward pointing orange triangle and a brown triangle, respectively. The positions of the Ni and Te atoms at the start of the MD run are superimposed over the Na trajectory.

The dynamics of Na^+^ in the *P*6_3_/*mcm* and *P*6_3_22 structures of Na_2+*x*_Ni_2-*x*/2_TeO_6_ compositions of *x* = 0, 0.25, and 0.5 were studied with molecular dynamics at 500 K with the MACE-MP-0 ML-FF. √2*a* × √2*b* supercells were created of the lowest energy Na_2_Ni_2_TeO_6_ and Na_2.5_Ni_1.75_TeO_6_ structures, to give the same geometry as the lowest energy Na_2.25_Ni_1.875_TeO_6_ structure. After an equilibration period of 20 ps, the diffusion coefficient was calculated from the mean square displacement (MSD) for each structure over 100 ps, with the result shown in Figure 5. Raw MSD plots for each structure are shown in Figure S8.

For each composition *x*, the diffusion coefficient of Na^+^ is higher in the *P*6_3_22 structure relative to the *P*6_3_/*mcm*. As the *x* composition increases, the diffusion coefficient decreases, which is related to increased Na–Na interactions within the Na layer, as observed previously.^37^ The results highlight that even at a composition of x = 0.25, the Na stuffed *P*6_3_22 Na_2.25_Ni_1.85_TeO_6_ structure has a higher diffusion coefficient than the *P*6_3_/*mcm* Na_2_Ni_2_TeO_6_ structure at a composition of *x* = 0.

Analysis of the Na^+^ trajectories in *P*6_3_/*mcm* Na_2_Ni_2_TeO_6_ at 500 K (Figure 5b) shows that diffusion is predicted to occur mainly between the Na1 and Na2 sites, with limited occupation of the Na3 sites, consistent with previous reports.^38^ In contrast, in the *P*6_3_22 Na_2.25_Ni_1.875_TeO_6_ structure (Figure 5c), there is significant hopping between all Na1, Na2 and Na3 sites, leading to exceptional long-range diffusion. The Na4 sites within the Ni–Te layer remained immobilized in all Na-excess structures throughout the simulation. The findings will be further discussed in the context of the solid-state NMR results below.

### Solid-State Nuclear Magnetic Resonance Spectroscopy

**Figure 6:**
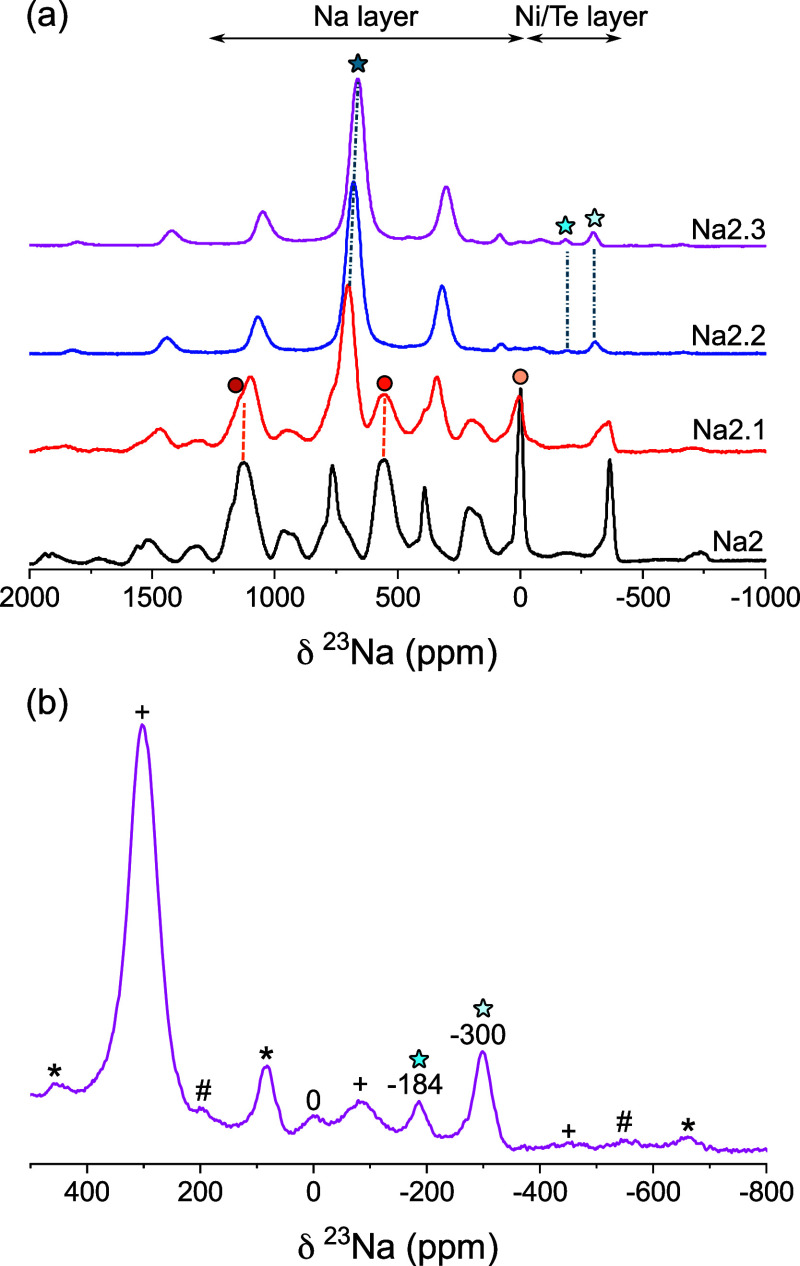
(a) ^23^Na MAS NMR spectra of Na_2+*x*_Ni_2-*x*/2_TeO_6_ phases from *x* = 0 – 0.3. All spectra were acquired at a spinning speed of 30 kHz. Circles and stars indicate the isotropic peaks in the *P*6_3_/*mcm* to the *P*6_3_22 structures, respectively. Dashed lines are included to show the evolution of the isotropic peaks between spectra. All spectra are scaled to a maximum peak intensity of unity. (b) Zoomed in ^23^Na NMR spectrum of the Na_2.3_Ni_1.85_TeO_6_ phase, showing the isotropic peaks at negative frequencies. The shifts of the isotropic resonances (in ppm) are given above the peaks. Spinning sidebands are indicated with symbols (*, + , #).

The local arrangement of Na sites as a function of Na excess in Na_2+*x*_Ni_2-*x*/2_TeO_6_ was further studied with ^23^Na solid state NMR (Figure 6). For the Na_2_Ni_2_TeO_6_ end member, three main isotropic resonances (circles) are observed in Figure 5a at 1125, 552 and −3 ppm. To confirm that the remaining peaks in the spectrum were due to spinning sidebands, spectra were acquired at slower spinning speeds (Figure S9) and with a 2D pj-MATPASS pulse sequence (Figure S10). In addition to the main peaks, small shoulder peaks were also observed on around the main resonances in the pj-MATPASS spectrum at −59, 92, 564, 717, 1152, and 1286 ppm. As the Na-excess concentration increased to Na_2.1_Ni_1.95_TeO_6_, additional peaks (stars) are observed in Figure 6a at 701 and ∼ −320 ppm. The 3 peaks previously observed in Na_2_Ni_2_TeO_6_ are still present with decreased intensity. At Na_2.2_Ni_1.9_TeO_6_, the original peaks are absent, leaving a single, intense resonance at 678 ppm, with 2 smaller peaks at −307 and −193 ppm. Increase in the Na-excess to Na_2.3_Ni_1.85_TeO_6_ results in a small shift in the dominant peak to 661 ppm. The reduction in the shift is due to the reduction in the number of paramagnetic Ni^2+^ centers in the first and second coordination spheres of Na^+^. The peaks at −300 and −184 ppm are still present with increased intensity Figure 6b. A small peak at 0 ppm is also observed, which accounts for less than 1% of the total intensity. This peak is tentatively assigned to diamagnetic impurities on the surface of the cathode particles.

**Figure 7:**
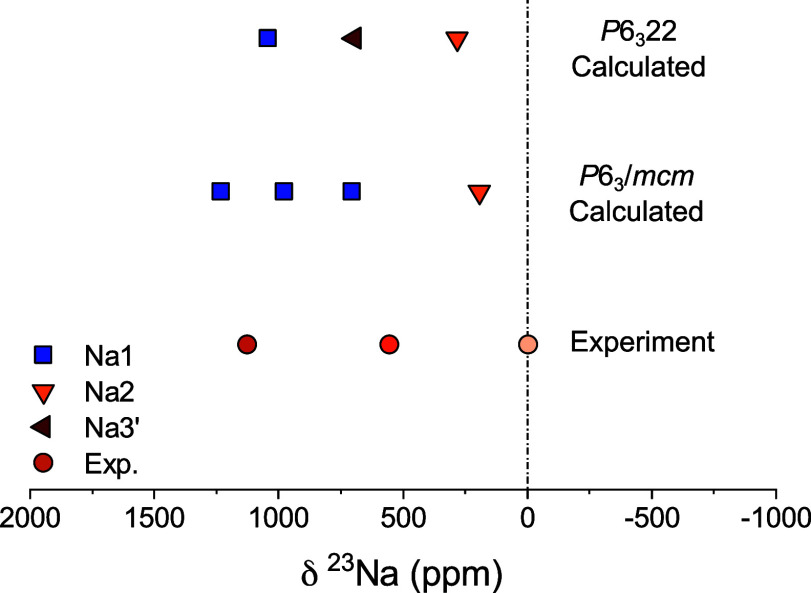
Comparison of experimental (Exp.) and DFT computed ^23^Na NMR shifts for the *P*6_3_/*mcm* and *P*6_3_22 structures of unsubstituted Na_2_Ni_2_TeO_6_. All computed shifts are scaled to 320 K using a spin of S = 1 and a Weiss constant of θ= −32 K.

To assign the peaks in Figure 6 to environments within the *P*6_3_/*mcm* and *P*6_3_22 phases observed via diffraction, first-principles calculations were used. The dominant interaction in solid-state ^23^Na NMR spectra of layered cathodes containing paramagnetic transition metals is typically the Fermi contact interaction.^4,39−46^ Readers are directed to these references for further details of the spin transfer mechanisms that dictate the sign and size of the ^23^Na Fermi contact shifts. The ^23^Na Fermi contact shifts for the lowest energy stoichiometric *P*6_3_/*mcm* and *P*6_3_22 structures predicted in the previous section were calculated with DFT as shown in Figure 7.

Large positive ^23^Na Fermi contact shifts are observed for the edge sharing Na1 sites in both the *P*6_3_/*mcm* (705, 976, and 1232 ppm) and *P*6_3_22 (1041 ppm) structures in Figure 4 (blue squares). Three distinct shifts are seen for the Na1 sites in the *P*6_3_/*mcm* structure as these sites experience different local displacements and bond lengths in the optimized low energy supercell structure. The Na1 sites with shifts of 705 and 1232 ppm are displaced toward neighboring Na2 and Na3 sites, respectively, while the Na1 site with a shift of 976 ppm is located at the center of the site. The Na2 sites, which share faces with two Ni, lead to the lowest shifts in both the *P*6_3_/*mcm* (192 ppm) and *P*6_3_22 (282 ppm) structures. The Na3′ site in the *P*6_3_22 structure has an intermediate shift of 695 ppm.

With the aid of DFT calculated shifts, the intense peak 1125 ppm in the experimental Na_2_Ni_2_TeO_6_ NMR spectrum is assigned to a Na1 site in the *P*6_3_/*mcm* structure. The sensitivity of the calculated Na1 shifts to local site distortions suggests that the small shoulder peaks on either side of the Na1 peak in the pj-MATPASS spectrum can tentatively be assigned to Na1 sites in distorted environments. The exact mechanism of the Na site distortion cannot be identified, but it could be related to local Na^+^ ordering or the presence of stacking faults. The dominant peak at −3 ppm is tentatively assigned to Na^+^ in an Na2 site of *P*6_3_/*mcm* structure. The lower shift observed experimentally compared to DFT (192 ppm) may result from the impact of additional second order quadrupole shifts not included in the current DFT treatment and to the choice of DFT functional. Shoulder peaks are again observed for the Na2 site, suggesting that local distortions are also present for face sharing Na configurations. The experimentally observed resonance at 552 ppm is closest to the shift to the Na1 sites (705 ppm) within the DFT optimized *P*6_3_/*mcm* structure, which are displaced toward the Na2 sites. The shift of the 552 ppm is very close to the average of the shifts of the Na1 (1125 ppm) and Na2 (−3 ppm) where (1125 + 3)/2 = 561 ppm. This resonance may either relate to heavily distorted, static Na1 positions or to Na that is rapidly exchanging between neighboring Na1 and Na2 sites at a frequency more rapid than the frequency separation between the peaks. This rapid exchange has been shown to be a common phenomenon in other Na-positive electrode materials.^46^ Further variable temperature measurements would be required to probe the dynamics of this site exchange, but it is beyond the scope of this work. However, the presence of distinct Na^+^ sites within the Na layer of the *P*6_3_/*mcm* structure supports the idea that there is Na^+^-ordering within the material.

**Figure 8:**
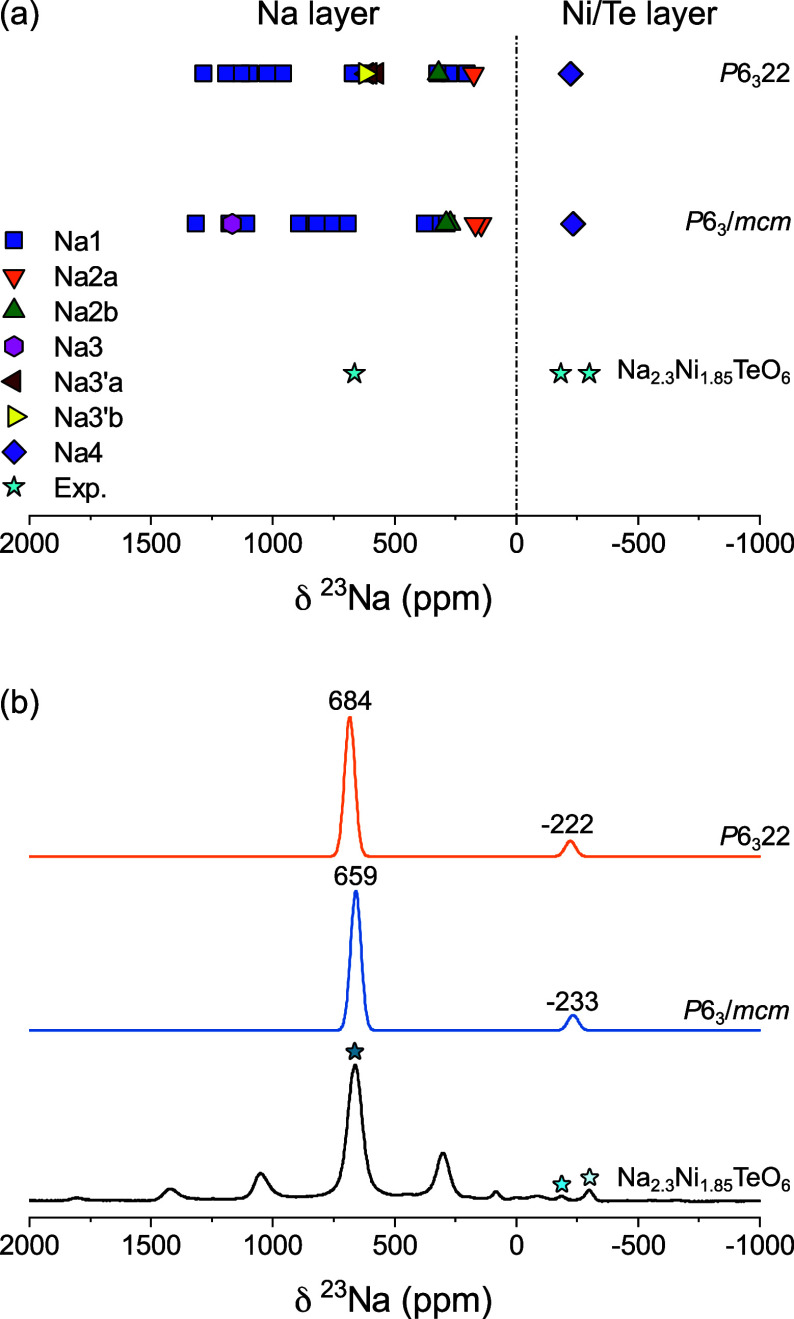
*(a)* Comparison of ^23^Na NMR shifts of compound Na_2.3_Ni_1.85_TeO_6_ (Exp.) with DFT computed shifts for lower energy Na-substituted *P*6_3_/*mcm* and *P*6_3_22 supercell structures (Na_20_Ni_14_Te_8_O_48_). (b) Comparison of experimental vs computationally predicted NMR spectra for *P*6_3_/*mcm* and *P*6_3_22 structures. A dynamic average of the shifts in the Na-layer was taken for computational spectra. The computational peaks are represented by a Gaussian with a width of 50 ppm. The isotropic resonances are indicated above the peaks in ppm. Isotropic peaks in the experimental spectrum are indicated with stars.

The ^23^Na NMR shifts of the lowest energy Na excess supercells (Figure 4a and b) were calculated as shown in Figure 8a. The inclusion of Na^+^ into the Ni/Te layer of the Na_20_Ni_14_Te_8_O_48_ (Na_2.5_Ni_1.75_TeO_6_) *P*6_3_/*mcm* and *P*6_3_22 structures results in a broad distribution of shifts, particularly for the Na1 (blue square) sites in Figure 8a. This broad distribution of shifts is due to different numbers of Ni^2+^–O-Na^+^ bond pathway contributions in the first and second coordination spheres of Na^+^ to the total Fermi shift. The introduction of Ni/Te layer Na^+^ (Na4 sites) leads to new, diamagnetic Na^+^(4)–O-Na^+^ bond pathway contributions, which do not contribute to the overall Fermi contact shift. Na2 sites still lead to the lowest shifts in the *P*6_3_/*mcm* and *P*6_3_22 structures, whereas Na3 sites in the *P*6_3_/*mcm* structure (1167 ppm) have larger shifts than Na3a’ (598–610 ppm) and Na3b’ (624 ppm) due to different neighboring configurations of Ni^2+^. Regardless of the site type, Figure 8(a) shows that all sites in the Na layer (Na1, Na2a, Na2b, Na3, Na3a’ and Na3b’) *P*6_3_/*mcm* and *P*6_3_22 structures have positive ^23^Na NMR shifts. In the experimental spectrum of Na_2.3_Ni_1.85_TeO_6_, only a single intense resonance is observed at 661 ppm. An average of the shifts for Na layer sites was taken for the *P*6_3_/*mcm* and *P*6_3_22 structures, resulting in shifts of 684 and 659 ppm. The shifts of both structures are in excellent agreement with the experimental value suggesting that all of the Na^+^ sites within the Na layer are being rapidly averaged. An exact assignment of the *P*6_3_/*mcm* or *P*6_3_22 structures is not possible based solely on the NMR, but as the *P*6_3_22 phase is the only phase observed by XRD at a composition of Na_2.3_Ni_1.85_TeO_6_, we can assign these Na sites to the Na layer of the *P*6_3_22 phase.

From the DFT computed shifts in Figure 8(b), the Na4 sites in the Ni/Te layer are the only environments that result in a negative shift for both the *P*6_3_/*mcm* (−233 ppm) and *P*6_3_22 (−222 ppm) structures. Similar negative ^7^Li NMR shifts are also observed for Li sites in the Ni layer of LiNiO_2_.^47^ The negative shifts in the current materials are also consistent with previously observed ^23^Na NMR shifts in the Na_3_Ni_1.5_TeO_6_ structure by the current authors.^3^ These sites only exhibit 3 Ni^2+^ neighbors along 90° Ni^2+^–O-Na bond pathways. The experimentally observed shift at −300 ppm is therefore assigned to the Na4 site in the Ni/Te layer of the *P*6_3_22 structure. Each 90° Ni^2+^–O-Na bond pathway contribution is therefore expected to contribute a shift of ∼ −100 ppm. The experimental shift of −184 ppm is assigned to a Na4 site with 2 Ni^2+^ neighbors and 1 diamagnetic Na^+^ neighbor. Na–Na first nearest neighbor connections were also observed previously in the Na_3_Ni_1.5_TeO_6_ structure.^3^ This result gives unambiguous evidence of the introduction of Na^+^ into the Ni/Te layer of the system which is accompanied by a disordering of the Na layer sites, leading to fast ionic motion in the as-synthesized materials.

### Electrochemical Characterization

**Figure 9:**
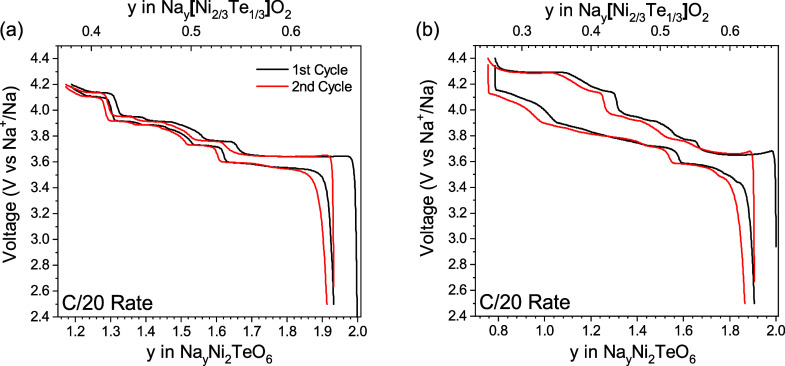
Voltage versus composition curves for P2-layered Na_2_Ni_2_TeO_6_ cycled in the voltage ranges of a) 2.5 to 4.2 V and b) 2.5 to 4.4 V versus Na^+^/Na. The bottom *x*-axis shows the total sodium content in the material. The top *x*-axis shows the sodium content within the sodium layer during cycling in accordance with the classical Na_*x*_MO_2_ layered formula. These cells were cycled against sodium metal with 1 M NaClO_4_ PC: FEC (9:1) (v:v) as the electrolyte.

With a firm understanding of the structure of these materials, the electrochemical properties could be characterized. Of particular interest is how the voltage-composition curve changes for the different P2-layered structures. Figure 9 shows the results for the P2-layered Na_2_Ni_2_TeO_6_ structure for the *P*6_3_/*mcm* space group. In the voltage range of 2.5 to 4.2 V versus Na^+^/Na, this phase shows numerous plateaus owing to the formation of intermediate ordered phases. These phases can be attributed to the face-sharing sites occupied by the Na^+^ in the interlayer space that share faces with either two Ni^2+^ or two Te^6+^. Widening the voltage range for cycling to 2.5 to 4.4 V reveals an additional reversible voltage plateau on charge at 4.3 V versus Na^+^/Na. When cycled to 4.4 V versus Na^+^/Na, the shape of the voltage curve on discharge changes to show fewer plateaus than when the material is only charged to 4.2 V versus Na^+^/Na. Moreover, the overpotential between the charge curve and discharge curve significantly increases in this case. The changing of the discharge voltage curve when cycled to higher voltage would suggest that there is less Na vacancy ordering during the intercalation process than the deintercalation at higher degrees of desodiation. The higher overpotential for the material when charged to above 4.3 V versus Na^+^/Na and the fewer observed discharge voltage plateaus may be related to an energy bottleneck for sodium reinsertion into the material owing to MO_2_ sheet gliding, as observed in other materials such as P2–Na_*x*_CoO_2_ system.^48^ Superstructure ordering and vacancy ordering in the sodium layer at low sodium content within the material may play a role in the formation of these metastable phases with a higher energy bottleneck for sodium reinsertion. Further in situ diffraction studies would be valuable to understand the specific nature of high voltage structural transformations in these materials, although it is beyond the scope of this study.

**Figure 10:**
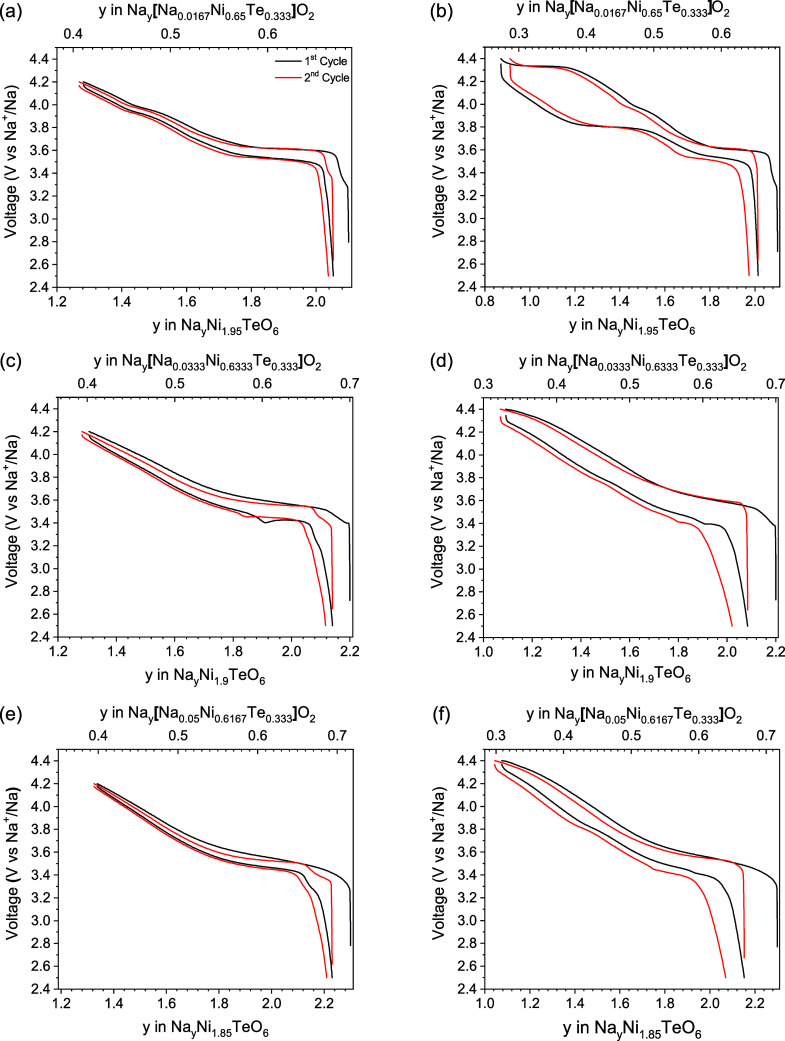
Voltage versus composition curves for P2-layered Na_2+*x*_Ni_2-*x*/2_TeO_6_ (0.1 ≤ *x* ≤ 0.3) cycled in the voltage range of a,c,e) 2.5 to 4.2 V and b,d,f) 2.5 to 4.4 V versus Na^+^/Na. The bottom *x*-axis shows the total sodium content in the material. The top *x*-axis shows the sodium content within the sodium layer during cycling in accordance with the classical Na_*x*_MO_2_ layered formula, assuming the sodium in the MO_2_ layer is not inserted/extracted during cycling. These cells were cycled at a rate of C/20 against sodium metal, with 1 M NaClO_4_ PC: FEC (9:1) (v:v) as the electrolyte.

The voltage versus composition curves of the Na_2+*x*_Ni_2-*x*/2_TeO_6_ (0.1 ≤ *x* ≤ 0.3) materials in the voltage ranges of 2.5 to 4.2 V and 2.5 to 4.4 V versus Na^+^/Na are shown in Figure 10. To allow for further comparison, plots of voltage vs specific capacity have been included in Figure S11. Even with a small amount of excess sodium for the Na_2.1_Ni_1.95_TeO_6_ material, the voltage curve is drastically different than that of the Na_2_Ni_2_TeO_6_ phase, indicating that significantly less sodium ordering is occurring in the interlayer space during the intercalation/deintercalation process. When charged to 4.2 V versus Na^+^/Na, Na_2.1_Ni_1.95_TeO_6_ shows an almost smooth voltage curve with a small hump around 3.9 V. However, when charged to 4.4 V versus Na^+^/Na, Na_2.1_Ni_1.95_TeO_6_ still shows a significant overpotential. Since the *P*6_3_/*mcm* P2-layered phase is the dominant phase in the Na_2.1_Ni_1.95_TeO_6_ composition, the high overpotential can be attributed to the sluggish kinetics of this P2-layer stacking. The reduction in the number of voltage plateaus in the voltage range of 2.5 to 4.2 V versus Na^+^/Na compared to the voltage curve of the stoichiometric Na_2_Ni_2_TeO_6_ shows the significance of the effect of a small amount of sodium occupation in the MO_2_ layer. The X-ray diffraction pattern shows that the *P*6_3_22 P2-layered phase is almost undetectable in the Na_2.1_Ni_1.95_TeO_6_ composition, the disruption of the vacancy ordering at low to intermediate levels of desodiation when charged to 4.2 V versus Na^+^/Na can be attributed primarily to the sodium in the MO_2_ layer. This result is consistent with the findings for the previously investigated O’3-Na_3_Ni_1.5_TeO_6_ material where it was shown that a small amount of sodium in the MO_2_ layer can be beneficial to the electrochemical performance of the material by disrupting the sodium vacancy ordering in the interlayer space. The fact that the sodium in the MO_2_ layer of P2–Na_2.1_Ni_1.95_TeO_6_ (Na_0.683_[Na_0.0167_Ni_0.65_Te_0.333_]O_2_) is a tenth of that in the O’3-Na_3_Ni_1.5_TeO_6_ (Na_0.8333_[Na_0.1667_Ni_0.5_Te_0.333_]O_2_) material gives insight to how little sodium is needed in the MO_2_ layer to disrupt the vacancy ordering in adjacent sodium layers.

As the sodium concentration in the as-prepared materials is increased beyond the Na_2.1_Ni_1.95_TeO_6_ composition, the electrochemical curve continues to improve. For the Na_2.2_Ni_1.9_TeO_6_ and Na_2.3_Ni_1.85_TeO_6_ materials, there is a single voltage plateau in both voltage ranges cycled. Additionally, the overpotential in these two materials is less than for the Na_2_Ni_2_TeO_6_ and Na_2.1_Ni_1.95_TeO_6_ materials when charged to 4.4 V versus Na^+^/Na, which is consistent with the hypothesis that the overpotential was being induced by strong Na^+^ ordering at high levels of desodiation. The lack of Na^+^ ordering for the Na_2.2_Ni_1.9_TeO_6_ and Na_2.3_Ni_1.85_TeO_6_ materials can be accounted for by three synergistic qualities of these two materials: the increasing amount of sodium in the MO_2_ layer, the change in the P2-layered MO_2_ superstructure from the *P*6_3_/*mcm* space group to the *P*6_3_22 space group, and the induced stacking faults in the *P*6_3_22 space group. Each of these consequences result from the substitution of Na^+^ for Ni^2+^in the MO_2_ layer, which is a novel finding for a P2-layered Na_*x*_MO_2_ oxide. It is possible that the excess Na^+^ in the transition-metal layer delays the onset of the MO_2_ layer gliding during cycling, thus stabilizing the P2 stacking sequence. However, further investigation is required to validate this hypothesis. Additionally, further studies at higher cycling rates would be beneficial to explore the impact of Ni–Te layer Na^+^ on the kinetics of desodiated phases, as all electrochemical measurements in this work were performed at relatively slow cycling conditions (C/20) to study electrochemical processes near equilibrium.

In each of the materials in the Na_2-*x*_Ni_2-*x*/2_TeO_6_ (0 ≤ *x* ≤ 0.3) series, the amount of sodium inserted/extracted from the material does not exceed what can be accounted for by the Ni^2+/3+^ redox couple. As with other nickel tellurate compounds investigated as lithium or sodium electrode materials, the Ni^2+/3+^ redox couple for this material shows a potential higher than traditionally expected owing to the inductive effect induced by the strong Te–O covalent bonding in the TeO_6_ motif.

### General Discussion

Na-substitution into the transition metal layer is a significantly underexplored avenue for improving the electrochemical properties in layered Na cathode systems. This study demonstrates that this approach is possible for P2 layered systems, in addition to O3 layered systems, such as Na_3_Ni_1.5_TeO_6_ and Na_3+x_Ni_2–2__*x*_Fe_*x*_SbO_6_, as shown in previous works.^3,4^ An added benefit of the Na^+^ substitution strategy in the current P2 layered materials is that Na^+^ substitution for Ni^2+^ in the transition metal layer was accompanied by additional insertion of Na^+^ in the Na layer through charge balance, increasing the total capacity. The ability to introduce >10% Na excess into the P2 Na_2-*x*_Ni_2-*x*/2_TeO_6_ materials suggests that Na-substitution should be possible in a wide range of other P2 and O3 layered cathodes that are currently under investigation, particularly in systems where Ni^2+^ ions are available for substitution.

The results in this paper also highlight important lessons about the synthesis conditions of commonly studied layered Na_*x*_MO_2_ oxides. The results presented herein for the stoichiometric P2–Na_2_Ni_2_TeO_6_ phase are not in agreement with those previously obtained by Gupta et al. in their initial report.^8^ Closer inspection of the X-ray diffraction pattern of their electrochemically tested material shows peaks that can be attributed only to NiO, one of the precursor materials. It was explicitly stated that no excess sodium was used during their synthetic procedure to form the P2–Na_2_Ni_2_TeO_6_ material, but it is possible that reaction conditions did not allow for all the NiO to react and the resulting product responsible for the reported electrochemical performance was closer to a P2-layered Na_2.1_Ni_1.95_TeO_6_ composition. The initial discovery into this effect was born out of trying to reproduce the initial electrochemical results for the Na_2_Ni_2_TeO_6_ composition and not being able to obtain the proper voltage curve until excess sodium was used for synthesis, whereupon a NiO impurity developed–see Figure S12 (Supporting Information). The NiO impurity peaks in the X-ray diffraction pattern grew in intensity with greater excess of Na_2_CO_3_ used for synthesis. Additionally, a drastic shift in the superstructure region of the diffraction pattern occurred when 10% excess Na_2_CO_3_ was used for material synthesis. This result led to the hypothesis that sodium was replacing nickel within the nickel–tellurium layer of the material and spurred the full investigation that we have presented.

The addition of excess Na_2_CO_3_ is commonly adopted in the lab scale synthesis of other layered Na_*x*_MO_2_ cathode materials, which are often required to suppress the formation of secondary phases. It is possible that the improvements in electrochemistry of some of these materials may come from small amounts of Na^+^ substitution into the transition metal layers in addition to an increase in the phase purity. Careful local structure characterization through techniques such as solid-state NMR coupled with first-principles calculations, is therefore highly recommended for studies of new Na_*x*_MO_2_ cathode systems to assess the potential role of Na^+^ substitution on improving electrochemical performance.

## Conclusion

The P2- layered Na_2+*x*_Ni_2-*x*/2_TeO_6_ (0 ≤ *x* ≤ 0.5) system provides unique insight into the nuances of layered Na_*x*_MO_2_ oxide cathodes for sodium-ion batteries with a rich dynamic between composition, structure, and electrochemical profile. The substitution of Na^+^ for Ni^2+^ allows for the disruption not only of the intralayer ordering in the MO_2_ layer, but also of the Na^+^ ordering in the adjacent layers. Additionally, the MO_2_ layer-stacking shifts beyond the Na_2.1_Ni_1.95_TeO_6_ composition such that the TeO_6_ octahedra in adjacent MO_2_ layers no longer stack on top of each other, contributing to the lack of Na^+^ ordering owing to competing electrostatic repulsion from Te^6+^. These factors contribute to the P2 - Na_2.2_Ni_1.9_TeO_6_ and Na_2.3_Ni_1.85_TeO_6_ materials having voltage-composition curves with a low overpotential that are reminiscent of commercial layered LiMO_2_ oxides and show no evidence of Na^+^ ordering or MO_2_ layer gliding when charged 4.4 V versus Na^+^/Na.

The findings of this study reiterate the care that must be taken when synthesizing Na_*x*_MO_2_ layered oxides as the sodium concentration in the material can have a drastic effect on its resulting structure and electrochemical performance. In this case for the Na_2+*x*_Ni_2-*x*/2_TeO_6_ materials, this effect is beneficial toward their electrochemical performance. In addition to further investigation into the effect of sodium substitution for M-ions in the MO_2_ layer of Na_*x*_MO_2_ layered oxides toward superior electrochemical performance, other layered Na_*x*_MO_2_ oxide systems may need to be revisited if their synthesis route has required excess sodium to ensure that the assumed final composition and electrochemical properties of the material are accurate.

## Supporting Information Available

The Supporting Information file contains Le Bail X-ray refinements, pj-MATPASS NMR spectra, voltage vs capacity plots and additional basin hopping and molecular dynamics simulations of the Na_2.25_Ni_1.875_TeO_6_ structure (PDF)

## Dedication

^#^ J.B.G. deceased on the 25th of June 2023.
