# Supplementary material for: Understanding the Beneficial Role of Transition-Metal Layer Na+ Substitution on the Structure and Electrochemical Properties of the P2-Layered Cathode Na2+xNi2–x/2TeO6
Source: Chem Mater. 2025 Mar 13;37(9):3040–53. doi: 10.1021/acs.chemmater.4c02798 (PMC12079799; doi:10.1021/acs.chemmater.4c02798)
Supplement: Supplementary file 1 — cm4c02798_si_001.pdf [file cm4c02798_si_001.pdf]

Supporting Information

Understanding the Beneficial Role of Transition-Metal Layer Na^+ Substitution on the Structure and Electrochemical Properties of the P2-Layered Cathode $Na_{2+x}Ni_{2-x/2}TeO_6$

Nicholas S. Grundish[1]*, Graeme Henkelman[2], John B. Goodenough[1#], Claude Delmas[3], Dany Carlier[3,4], and Ieuan D. Seymour[2,5]*

[1]Materials Science and Engineering Program and Texas Materials Institute, University of Texas, Austin, TX 78712, USA

[2] Department of Chemistry and Oden Institute for Computational Engineering and Sciences, The University of Texas at Austin, Austin, Texas 78712, United States

[3] Univ. Bordeaux, CNRS, Bordeaux INP, ICMCB, UMR 5026, F-33600 Pessac, France

[4] RS2E, Réseau Français sur le Stockage Electrochimique de l'Energie, FR CNRS 3459,F-80039 Amiens Cedex 1, France

[5] Advanced Centre for Energy and Sustainability, Department of Chemistry, School of Natural and Computing Sciences, University of Aberdeen, Aberdeen, AB24 3FX, UK

*e-mail: nicholas.grundish@utexas.edu; ieuan.seymour@abdn.ac.uk

J. B. G. deceased on the 25th of June 2023.

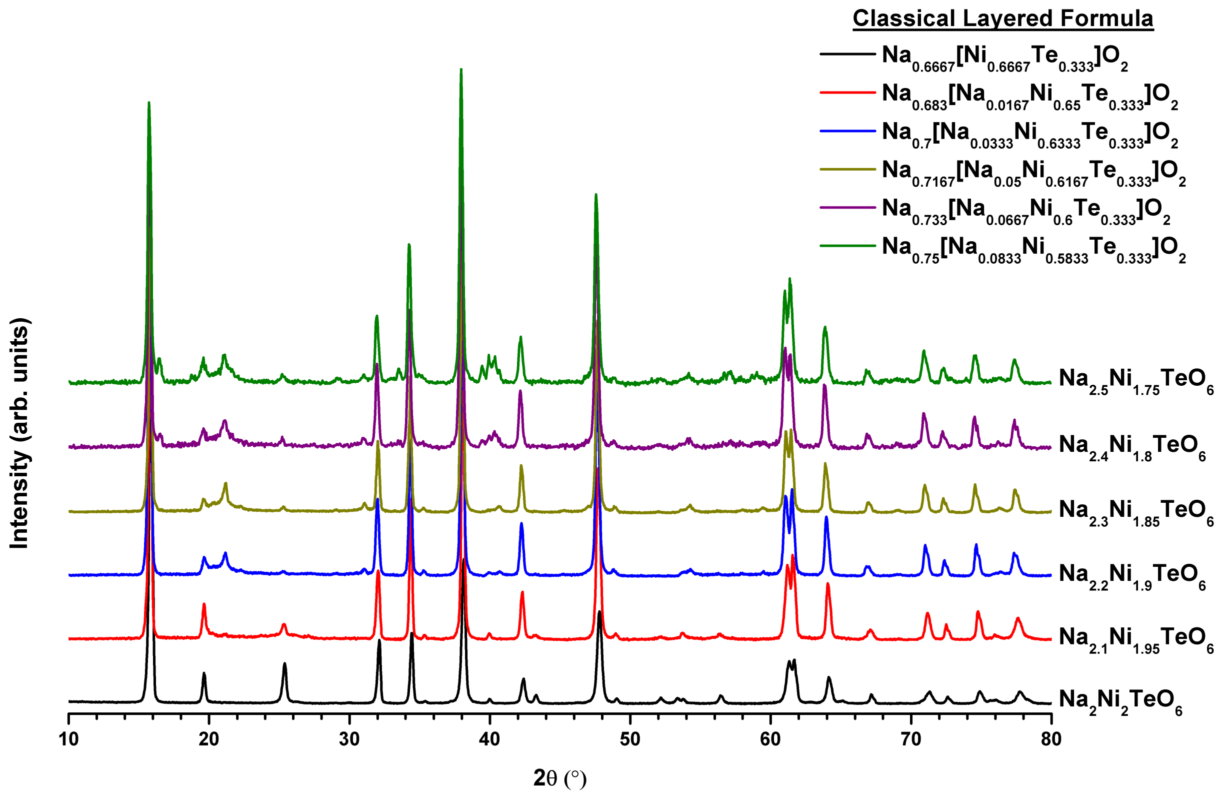

Figure S1. Powder X-ray diffraction patterns for $Na_{2+x}Ni_{2-x/2}TeO_6$ ($0 \leq x \leq 0.5$). The classical layered formula for each material is provided for reference.

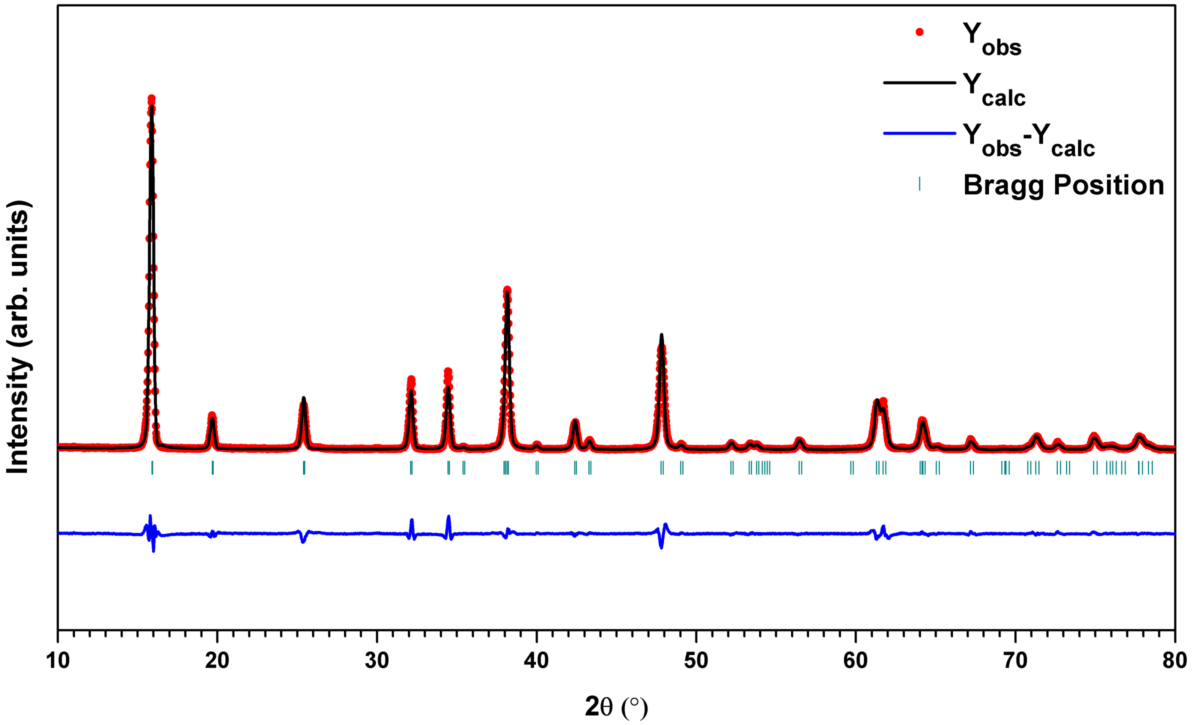

Figure S2. Le Bail refinement of the $Na_2Ni_2TeO_6$ powder X-ray diffraction pattern. The unit cell parameters and reliability factors from this refinement are summarized in Table 1. This pattern was fit with only the $P6_3/mcm$ space group.

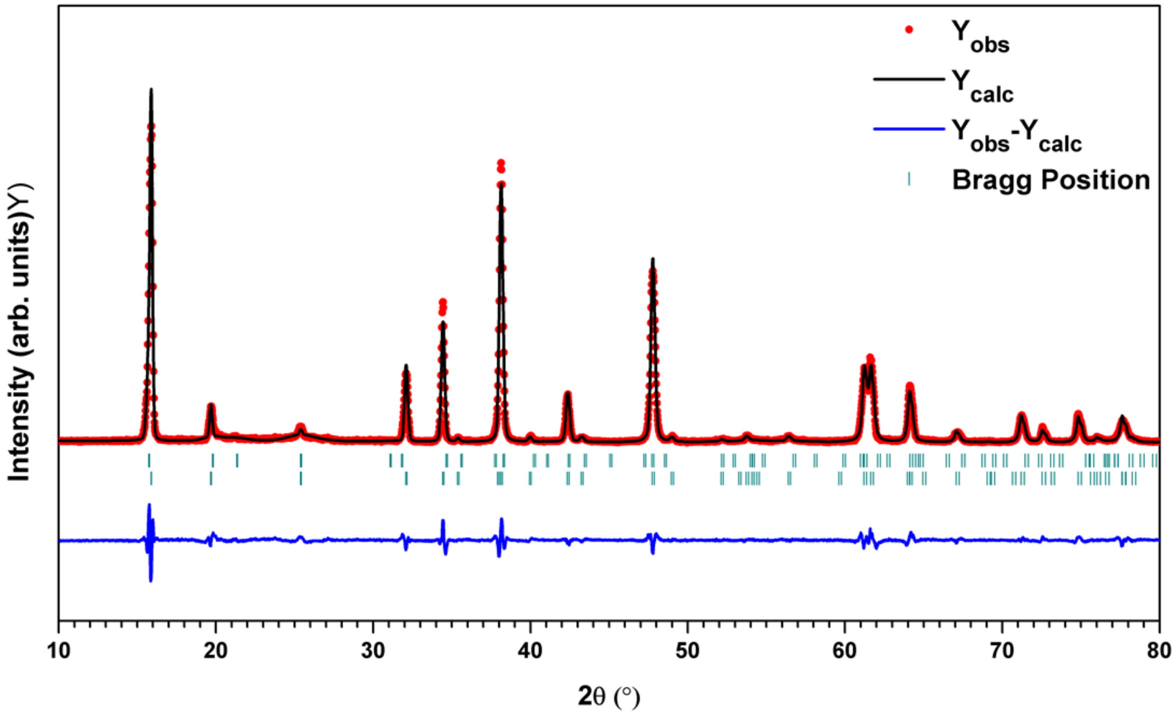

Figure S3. Le Bail refinement of the $Na_{2.1}Ni_{1.95}TeO_6$ powder X-ray diffraction pattern. The unit cell parameters and reliability factors from this refinement are summarized in Table 1. This pattern was fit with only the *P*6₃/*mcm* and *P*6₃22 space groups.

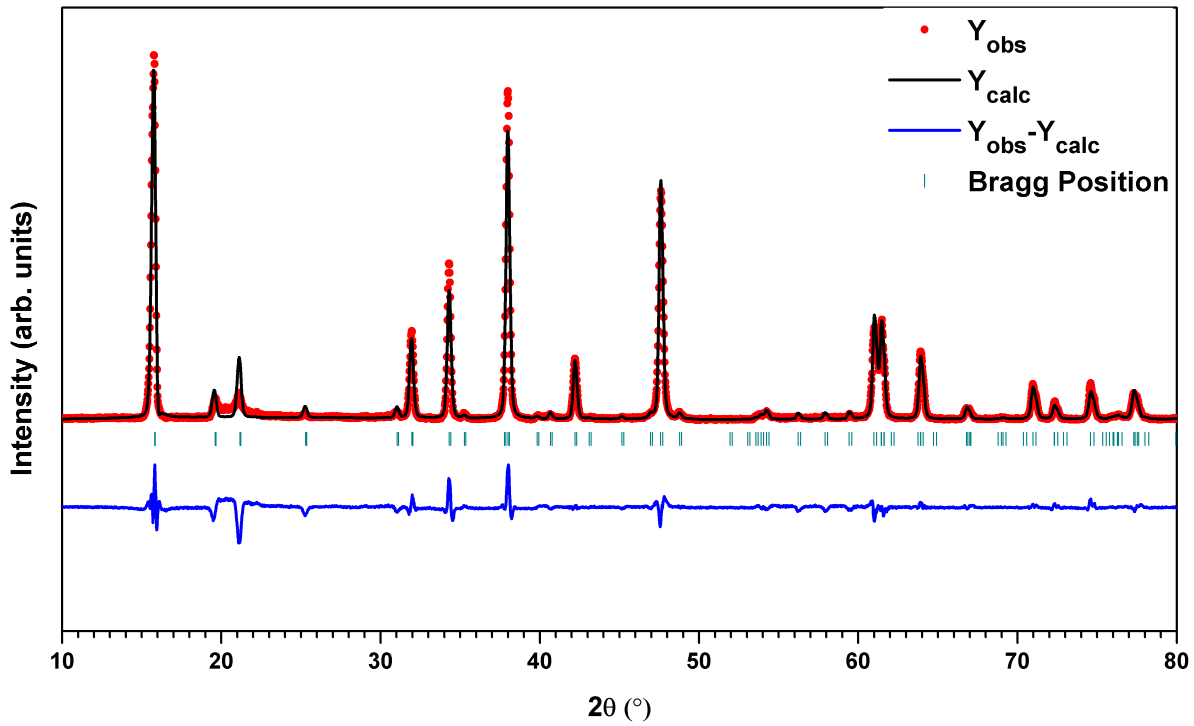

Figure S4. Le Bail refinement of the $Na_{2.2}Ni_{1.9}TeO_6$ powder X-ray diffraction pattern. The unit cell parameters and reliability factors from this refinement are summarized in Table 1. This pattern was fit with only the $P6_322$ space group.

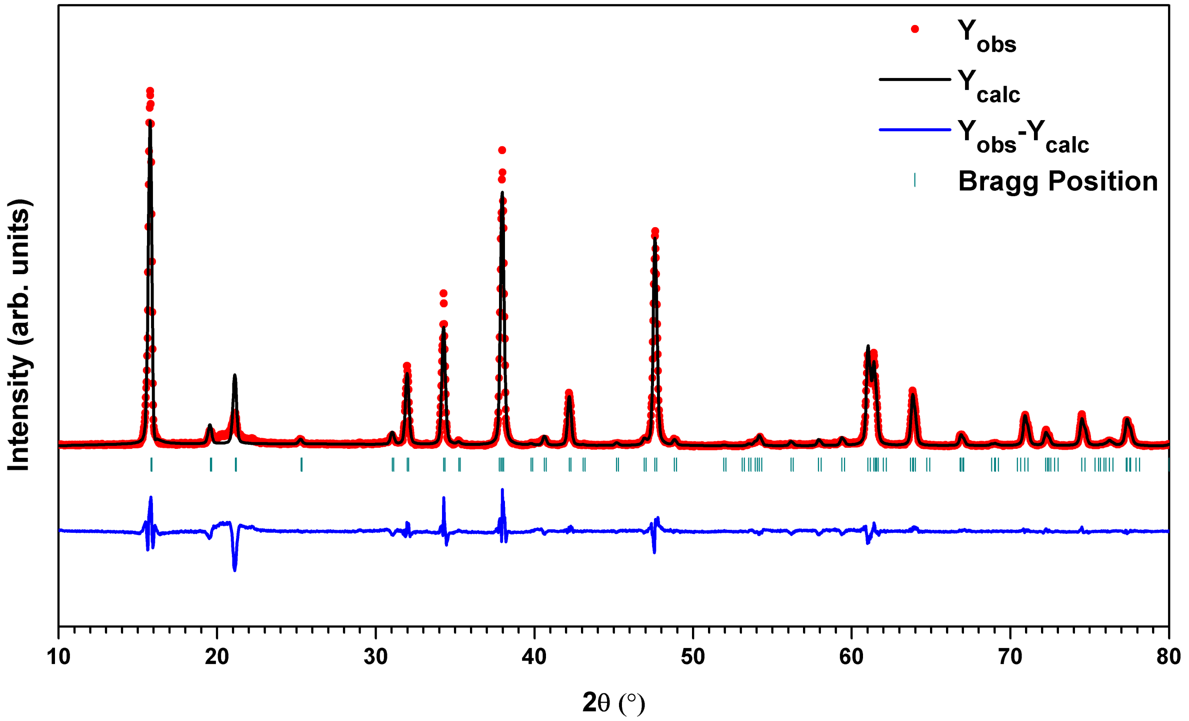

Figure S5. Le Bail refinement of the $Na_{2.3}Ni_{1.85}TeO_6$ powder X-ray diffraction pattern. The unit cell parameters and reliability factors from this refinement are summarized in Table 1. This pattern was fit with only the $P6_322$ space group.

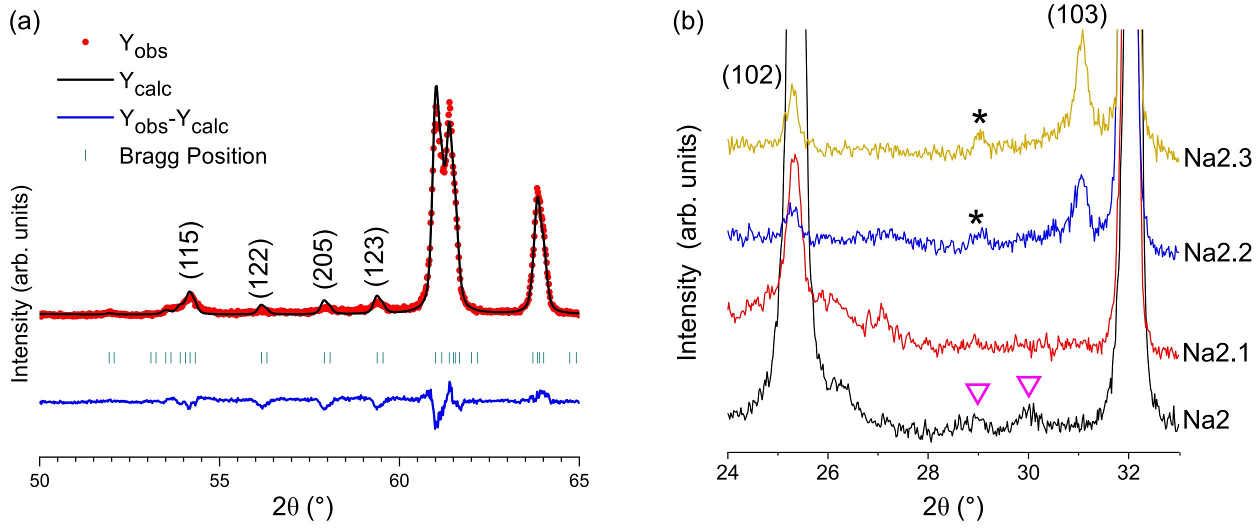

Figure S6. (a) Enlarged region from 24-33° 2θ of Le Bail refinement of the $Na_{2.3}Ni_{1.85}TeO_6$. The Miller indices of broaden peaks due to stacking faults are shown. (b) Enlarged X-ray diffraction patterns for $Na_{2+x}Ni_{2-x/2}TeO_6$ ($0 \le x \le 0.3$) in the region from 24-33° 2θ. Additional peaks in the *P*6$_3$/*mcm* $Na_2Ni_2TeO_6$ structure potentially associated with Na /vacancy ordering are shown with pink triangles. A single additional peak in the *P*6$_3$22 $Na_{2.2}Ni_{1.9}TeO_6$ and $Na_{2.3}Ni_{1.85}TeO_6$ structures is shown with an asterisk.

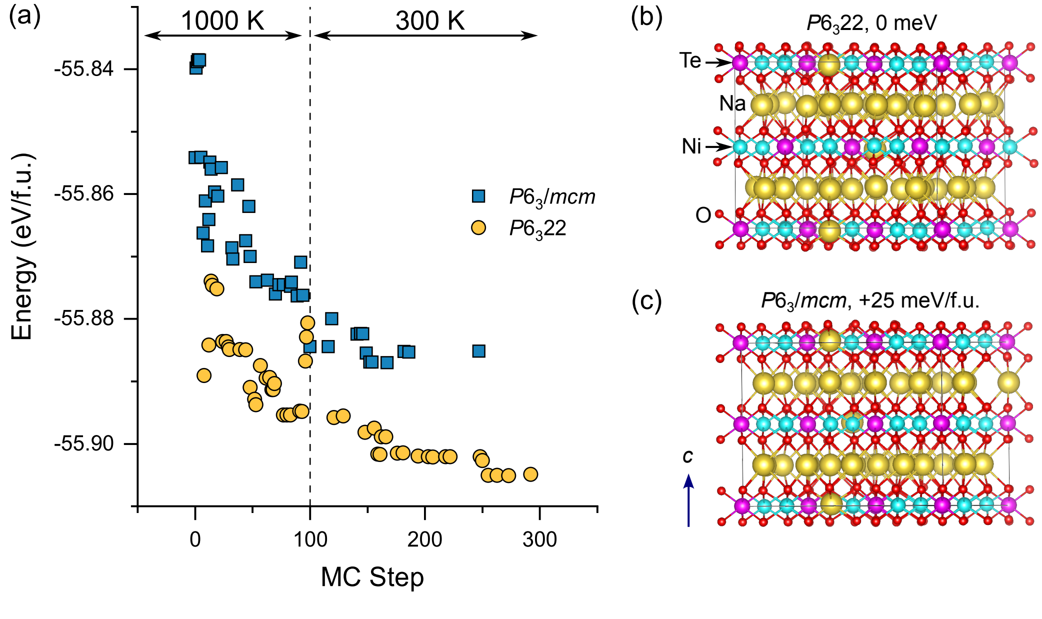

Figure S7. (a) MACE-MP-0 energetics of stoichiometric *P*6$_3$/mcm and *P*6$_3$22 structures with Monte Carlo basin hopping Na/V_{Na} swapping step. Only the energies of 'accepted' structures are shown. Dashed lines indicate different temperature regions. After 100 MC steps at 1000 K under fixed cell conditions, the lowest energy structure found was fully optimized and used for the subsequent 300 K steps in which the atomic positions and cell parameters were optimized at every

step. (b) and (c) DFT optimized supercell structures of lowest energy $P6_322$ and $P6_3/mcm$ structures, respectively, found at 300 K with MC. Relative energy difference between the structures using tight DFT+D3 parameters is shown.

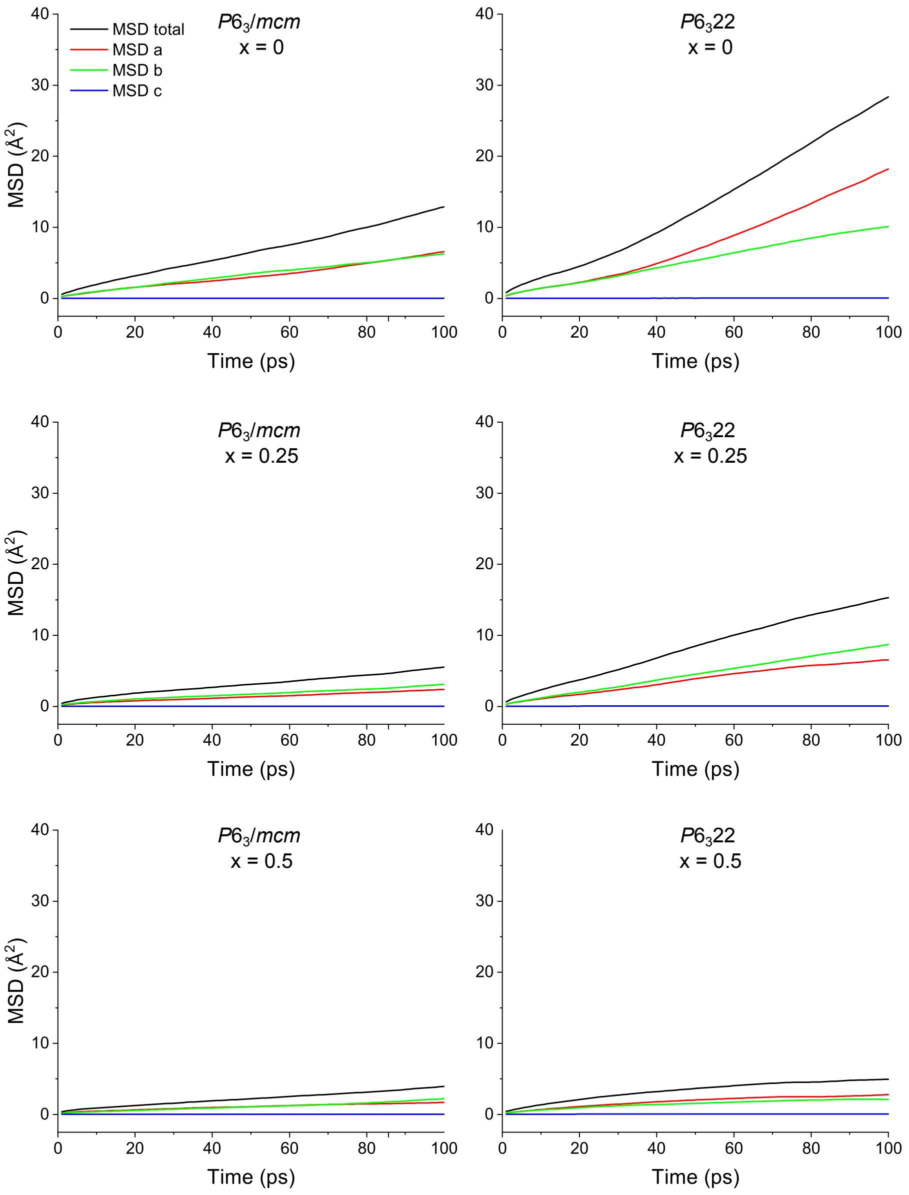

Figure S8. Plots of mean square displacement (MSD) vs time from molecular dynamics simulations of $Na_{2+x}Ni_{2-2/x}TeO_6$ supercells at 500 K. The MACE-MP-0 machine learning force field was used for all calculations.

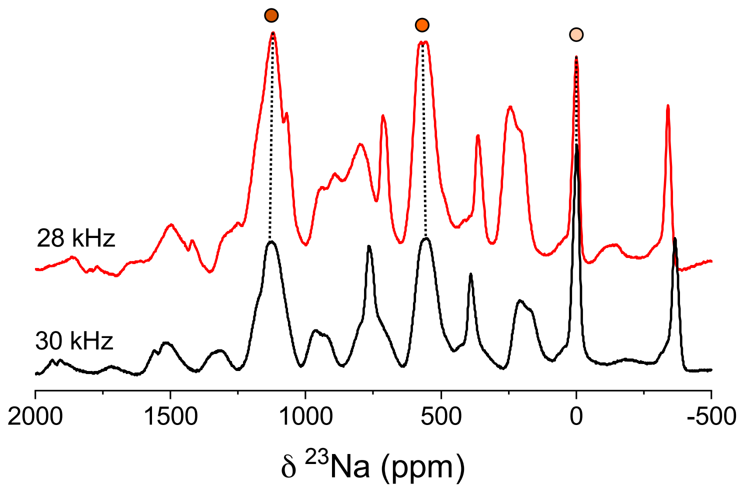

Figure S9. ^{23}Na MAS NMR spectra of $Na_2Ni_2TeO_6$ acquired at magic angle spinning speeds of 28 and 30 kHz. The three isotopic resonances are labelled with circles and dotted lines.

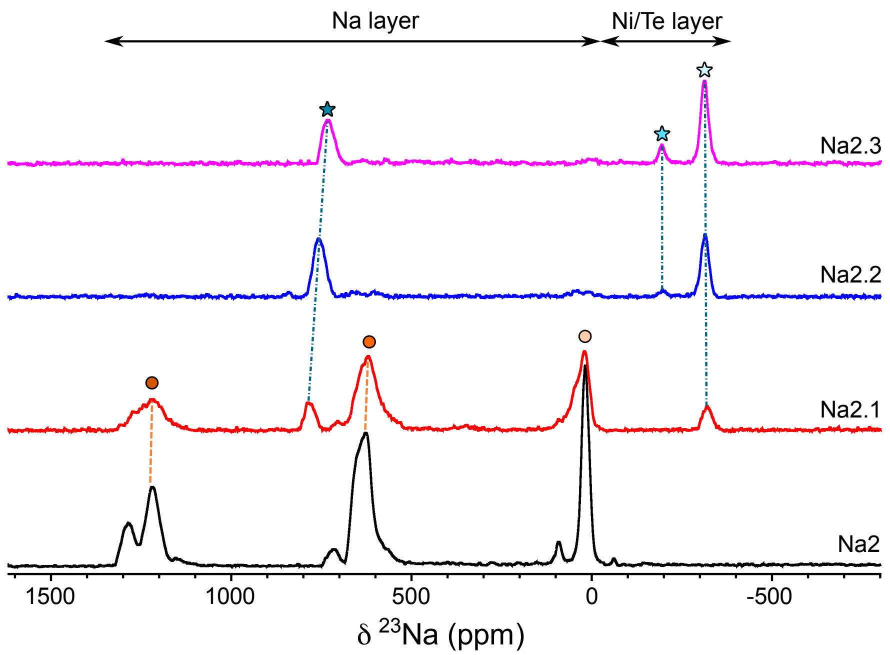

Figure S10. ^{23}Na pj-MATPASS spectra of $Na_{2+x}Ni_{2-x/2}TeO_6$ materials acquired at 8 kHz MAS. The 1D spectra showing only isotropic resonances for each material is produced by shearing the 2D pj-MATPASS spectrum along the F2 direction, and summing the spectra along F1.[1] Circles and stars indicate the isotropic peaks in the *P*6_3/*mcm* to the *P*$6_3$22 structures, respectively. Dashed lines are included to show the evolution of the isotropic peaks between spectra. A recycle delay of 0.01 s was used for the $Na_2Ni_2TeO_6$ material, whereas a recycle delay of 0.1s was used for $Na_{2.1}Ni_{1.95}TeO_6$, $Na_{2.2}Ni_{1.90}TeO_6$ and $Na_{2.3}Ni_{1.9}TeO_6$ materials. The relative intensities of the individual spectra are arbitrarily scaled to allow for easy comparison of different resonances. The relative intensity of peaks within each spectrum are not quantitative due to loss of signal during the pj-MATPASS sequence. The loss of signal is particularly evident for the fast-relaxing Na-layer environment the $Na_{2.3}Ni_{1.9}TeO_6$ system, which is significantly suppressed relative to the same environment in the quantitative Hahn-echo spectra in Figure 5 of the main text.

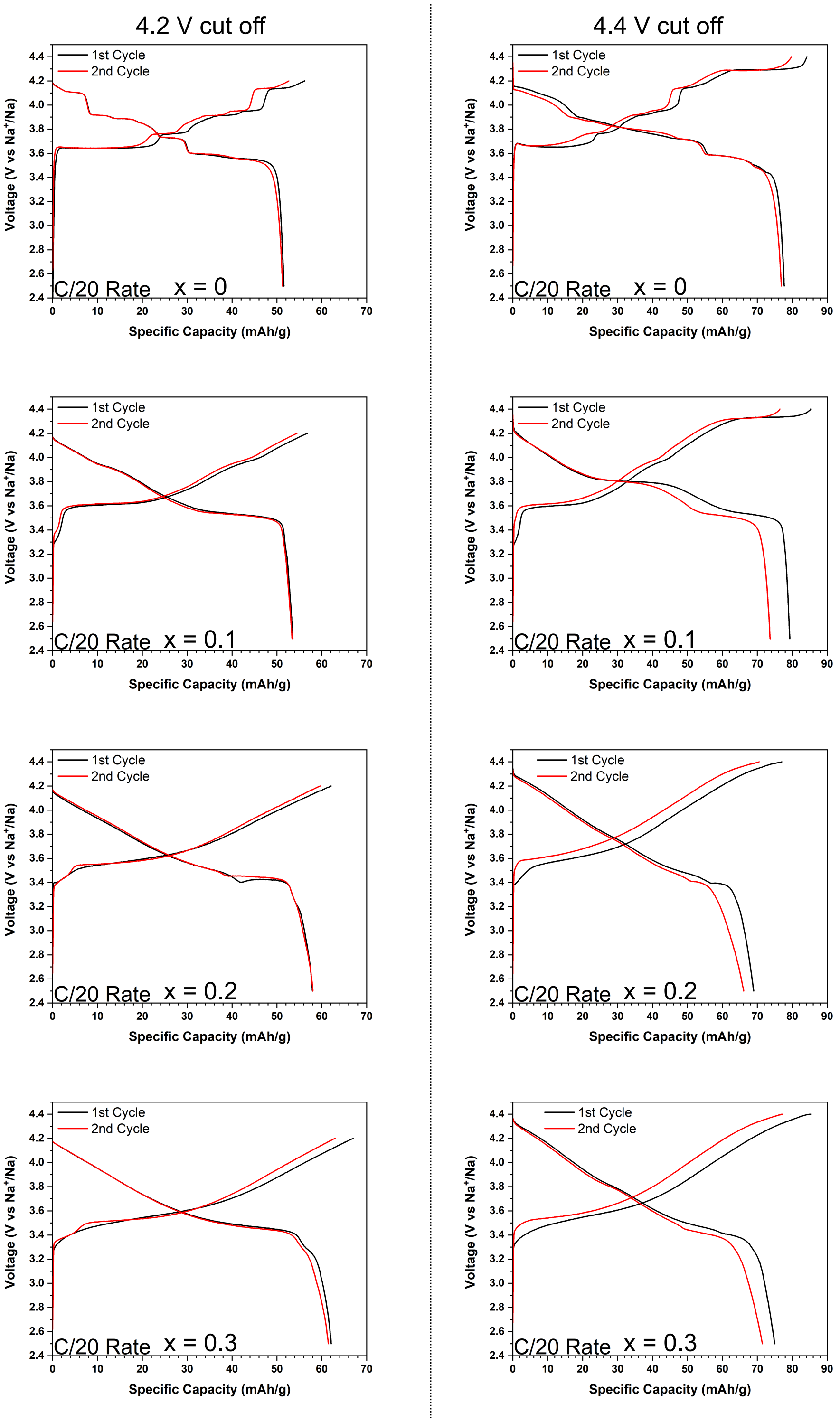

Figure S11. Voltage versus specific capacity curves for P2-layered $Na_{2+x}Ni_{2-x/2}TeO_6$ ($0.0 \leq x \leq 0.3$) cycled in the voltage range of 2.5 to 4.2 V (left) and 2.5 to 4.4 V (right) versus Na^+/Na. The same data plotted as a function of Na content are shown in Figures 9 and 10 of the main text. These cells were cycled against sodium metal with 1 M $NaClO_4$ PC: FEC (9:1) (v:v) as the electrolyte.

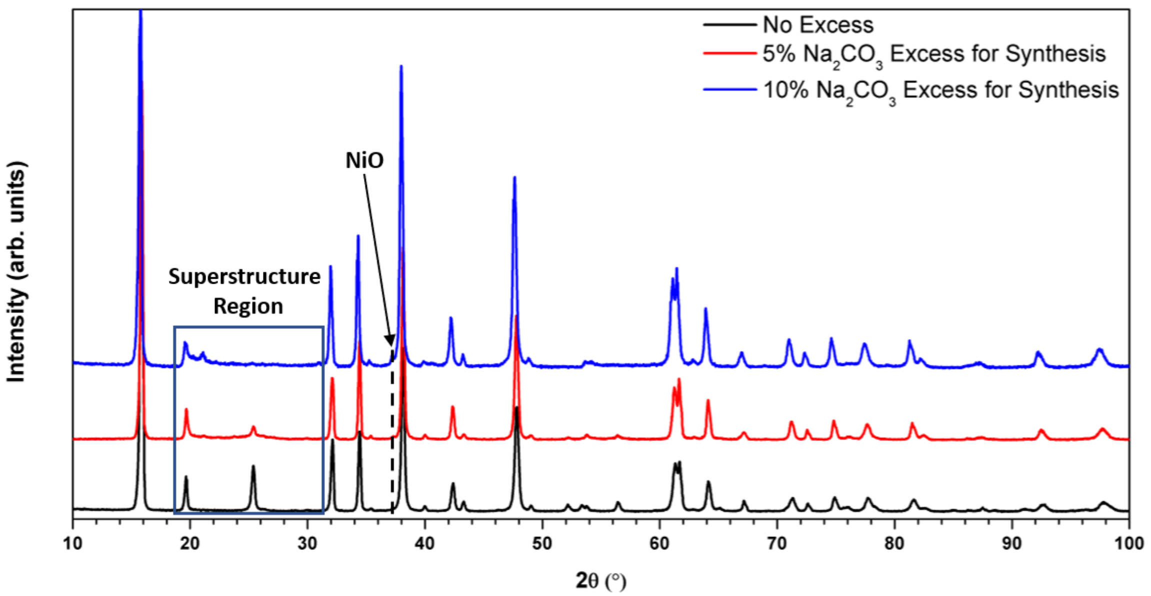

Figure S12. X-Ray Diffraction patterns of P2-layered $Na_2Ni_2TeO_6$ synthesized with varying amounts of sodium precursor.

References

(1) Hung, I.; Zhou, L.; Pourpoint, F.; Grey, C. P.; Gan, Z. Isotropic High Field NMR Spectra of Li-Ion Battery Materials with Anisotropy >1 MHz. *J. Am. Chem. Soc.* **2012**, *134* (4), 1898–1901. https://doi.org/10.1021/ja209600m.
